# Apoptotic Vesicle Membrane-Mediated Targeted Endothelial Mitochondrial Transplantation-Clearance Therapy for Diabetic Wound Healing

**DOI:** 10.34133/research.1042

**Published:** 2026-01-16

**Authors:** Zheyuan Hu, Shutong Qian, Bo Liao, Yuhuan Wang, Qian Lu, Jiayi Mao, Bolun Lu, Liucheng Zhang, Fei Wang, Danru Wang, Wenguo Cui, Xiaoming Sun

**Affiliations:** ^1^ Department of Plastic and Reconstructive Surgery, Shanghai Ninth People’s Hospital, Shanghai Jiao Tong University School of Medicine, Shanghai 200011, China.; ^2^ Department of Plastic Surgery, The First Affiliated Hospital, Zhejiang University School of Medicine, Hangzhou 310003, China.; ^3^ Department of Rehabilitation Medicine, Key Laboratory of Physical Medicine and Precision Rehabilitation of Chongqing Municipal Health Commission, The First Affiliated Hospital of Chongqing Medical University, Chongqing 400010, China.; ^4^ Department of Orthopaedics, Shanghai Key Laboratory for Prevention and Treatment of Bone and Joint Diseases, Shanghai Institute of Traumatology and Orthopaedics, Ruijin Hospital, Shanghai Jiao Tong University School of Medicine, Shanghai 200025, China.

## Abstract

Impaired mitophagy and the accumulation of damaged mitochondria are key drivers of endothelial cell (EC) dysfunction in diabetic wounds. While mitochondrial transplantation (MT) has demonstrated therapeutic potential in such mitochondrial damage-related diseases, its application is still thwarted by elusive mechanisms and practical hurdles such as poor targeting specificity and low delivery efficiency. Here, we reveal that MT acts by reactivating mitophagy to selectively eliminate dysfunctional mitochondria, thereby restoring mitochondrial homeostasis and rescuing EC functionality. To exploit this discovery, we engineer a biomimetic MT strategy through coating EC-derived apoptotic vesicle membrane (AVM) onto the surface of isolated mitochondria. The resulting mitochondria–AVM complex (Mito-AVM) leverages homologous targeting and phosphatidylserine-mediated “eat-me” signaling, achieving a remarkable 150% increase in delivery efficiency to ECs in diabetic wounds. Furthermore, we construct a 3-aminophenylboric acid-modified hyaluronic acid/polyvinyl alcohol hydrogel for the diabetic wound microenvironment, enabling reactive oxygen species/glucose-triggered sustained release of encapsulated Mito-AVM at the wound site. In summary, our work elucidates a fundamental mechanism of MT and provides an efficient and targeted strategy for MT therapy, offering fresh perspectives for diabetic wound treatment.

## Introduction

Endothelial cells (ECs) are pivotal in diabetic wound healing, with mitochondria serving as key signaling centers and energy sources [1,2]. However, in the diabetic environment of persistent hyperglycemia and oxidative stress, the mitochondria of ECs sustain severe damage, accompanied by a disruption of the mitochondrial quality control system—manifested as excessive fission, impaired fusion, and critically compromised mitophagy [3–5]. This results in the defective clearance and pathological accumulation of damaged mitochondria, which, in turn, amplifies reactive oxygen species (ROS) production, creating a vicious cycle of escalating mitochondrial injury [6]. Ultimately, these deficits impair EC migration and angiogenic capacity, critically hindering wound repair [7–9]. Consequently, the targeted clearance of damaged mitochondria emerges as a paramount therapeutic objective.

Current approaches to eliminate damaged mitochondria primarily rely on small-molecule drugs to induce mitophagy [10]. However, such approaches are often hampered by high cytotoxicity, off-target effects, and the risk of excessive mitochondrial depletion, limiting their clinical application [11]. Mitochondrial transplantation (MT) has emerged as a promising organelle-based therapy for diseases featuring mitochondrial damage [12–15]. For instance, MT into ischemic damaged tissues can enhance cellular metabolism and ameliorate disease progression [16,17]. Nonetheless, the therapeutic mechanism of MT remains inadequately explored. Existing research has predominantly focused on its capacity to boost oxidative phosphorylation (OXPHOS) levels in cells with high OXPHOS demand (e.g., cardiomyocytes and neurons), leaving its role in ECs with low OXPHOS demand poorly defined. Here, we demonstrate that MT enhances both metabolism and functionality of mitochondria-damaged ECs. However, this enhancement is not primarily manifested as increased OXPHOS level, but is instead characterized by augmented mitophagy. Mechanistically, recent research has demonstrated that MT exerts transient cytoprotective effects by degrading exogenous mitochondria, thereby stimulating mitochondrial biogenesis and biosynthesis in an EC engraftment model [18]. Although PINK1/Parkin-mediated mitophagy was similarly activated, MT did not lead to an increase in mitochondrial mass. Instead, it enhanced autophagic flux and promoted the selective clearance of endogenous damaged mitochondria. Thus, we hereby propose a novel mechanism: in mitochondria-damaged ECs, MT reactivates PINK1/Parkin-mediated mitophagy and increases autophagic flux to eliminate damaged mitochondria, thereby restoring the functionality of ECs.

**Fig. 1.**
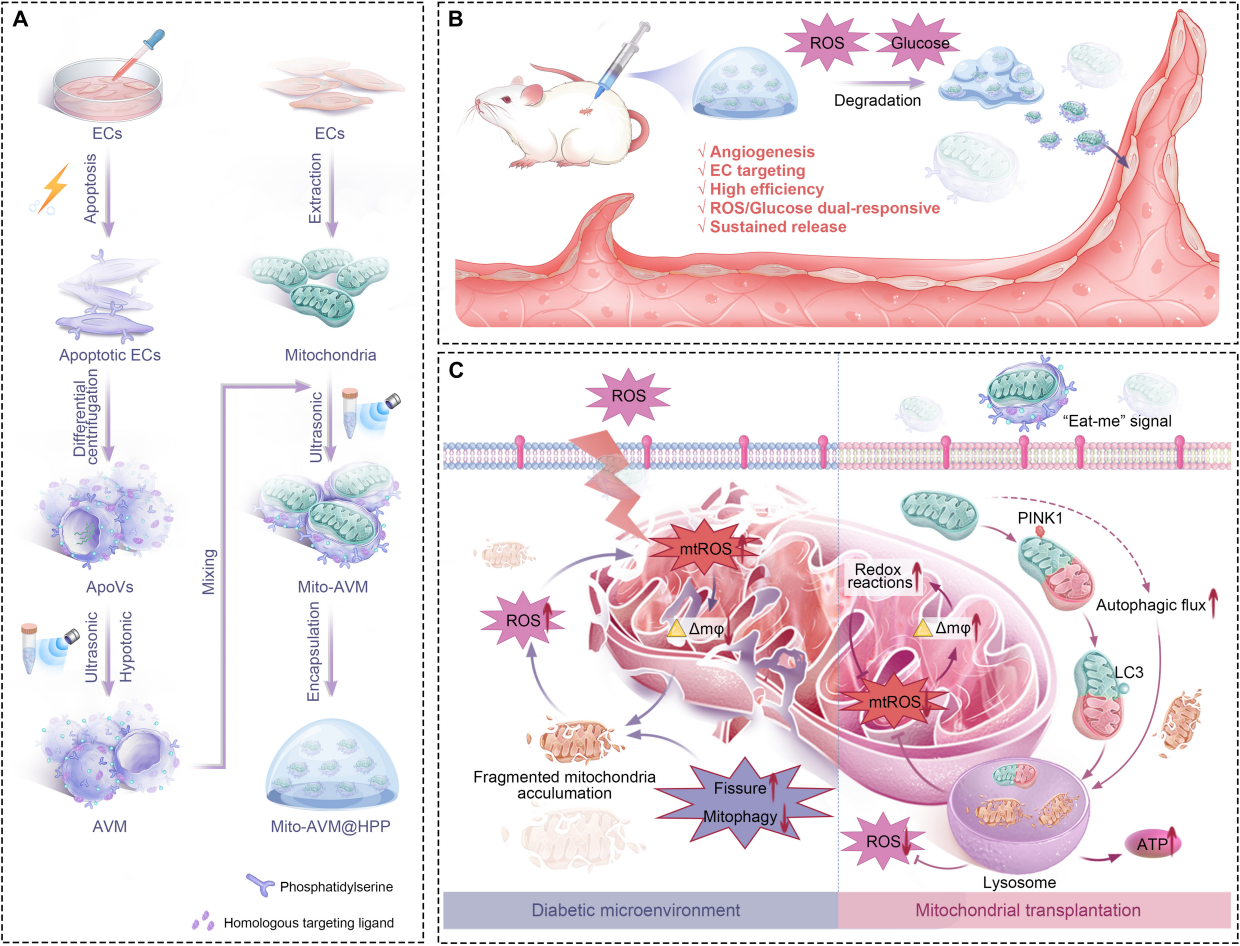
Schematic representation of the fabrication, application, and mechanism of Mito-AVM@HPP hydrogel. (A) The fabrication of Mito-AVM@HPP hydrogel targeted the mitochondrial transplantation-clearance system. The EC-derived ApoVs are processed into AVM, subsequently fused with mitochondria, and encapsulated in HPP hydrogel. (B) The application of Mito-AVM@HPP hydrogel in diabetic wounds. Mito-AVM@HPP hydrogel is injected onto the surface of diabetic wounds and continuously releases Mito-AVM in response to the high-glycemic, high-ROS environment. Mito-AVM targets ECs, resulting in highly efficient MT and the restoration of EC functionality (represented by vasculogenic capacity). (C) The mechanism by which Mito-AVM restores mitochondrial homeostasis and ECs function by reactivating mitochondrial autophagy.

Despite its promise, the clinical application of MT is constrained by poor targeting specificity, low delivery efficiency, and a narrow therapeutic window [15]. To overcome these barriers, we developed a biomimetic MT strategy utilizing apoptotic vesicle membrane (AVM). We engineered the mitochondria–AVM complex (Mito-AVM) by coating EC-derived AVM onto the surface of isolated mitochondria. This design leverages the homologous targeting ligands and the phosphatidylserine-mediated “eat-me” signal on the AVM surface to achieve high EC-targeting and delivery efficiency [19–23]. Subsequently, the Mito-AVM was loaded into an injectable, self-healing hydrogel formed by crosslinking 3-aminophenylboric acid-modified hyaluronic acid (HA-PBA) and polyvinyl alcohol (PVA), which exhibited dual responsiveness to ROS and glucose, enabling on-demand release of Mito-AVM in the diabetic wound microenvironment. Our hypothesis posits that Mito-AVM released from HA-PBA/PVA (HPP) hydrogels targets ECs for high-efficient MT, reactivates mitophagy to clear damaged mitochondria, restores mitochondrial homeostasis, and ultimately restores ECs function (Fig. 1). Consistent with this, we observe that the Mito-AVM exhibits more than double the EC uptake efficiency of free mitochondria both in vivo and in vitro. Additionally, the application of Mito-AVM loaded into HPP (Mito-AVM@HPP) hydrogel has the best effect on promoting diabetic wound healing, with the highest number of new blood vessels and the most mature collagen deposition in the wound tissue. This novel targeted MT strategy effectively clears damaged mitochondria in ECs, offering a promising new platform for the treatment of diabetic chronic wounds.

## Results and Discussion

### MT enhances mitochondria-damaged EC viability and functionality

**Fig. 2.**
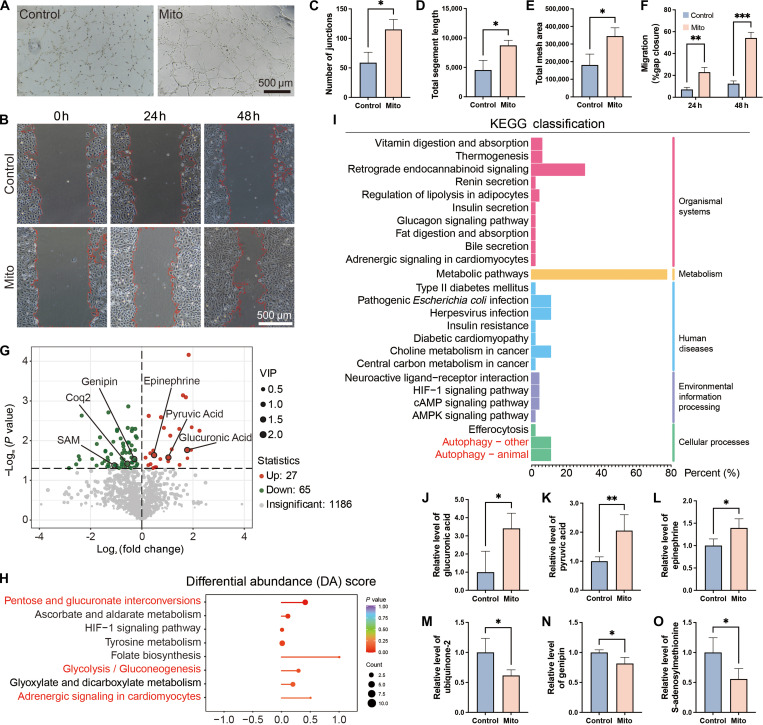
MT enhances mitochondria-damaged EC viability and functionality. (A) Tube formation assay. (B) Wound healing migration assay. (C) Number of junctions of new blood vessels. (D) Total segment length. (E) Total mesh area. (F) Relative migrated cell area. (G) Volcano plot of differentially expressed metabolites. (H) Differential abundance (DA) score bubble chart of KEGG pathways. (I) KEGG classification. (J) Relative level of glucuronic acid. (K) Relative level of pyruvic acid. (L) Relative level of epinephrine. (M) Relative level of ubiquinone2. (N) Relative level of genipin. (O) Relative level of SAM. Scale bars: 500 μm. Data are presented as mean ± SD, *n* ≥ 3. *P < 0.05, **P < 0.01, ***P < 0.001.

To simulate the chronic oxidative stress within the diabetic microenvironment and its damaging effects on EC mitochondria, we pretreated the cells with 100 μM H_₂_O_₂_ for 24 h. This pretreatment regimen exposes ECs to an oxidative environment that approximates the extracellular H_₂_O_₂_ levels found in diabetic chronic wounds. This condition effectively induces mitochondrial damage and cellular dysfunction while avoiding the induction of excessive apoptosis [24]. To ascertain the potential of MT in rescuing mitochondria-damaged ECs, we added mitochondria derived from healthy ECs to the damaged ECs at different mitochondrial number-to-cell number ratios (control; 10:1; 30:1; 100:1; 250:1; 500:1). After 24 h, MT at a 100:1 ratio significantly enhanced EC viability compared to controls (Fig. S1A), while also elevating normalized ATP level (Fig. S1B), improving vasculogenic capacity (Fig. 2A and C to E), and increasing migratory potential (Fig. 2B and F). It is noteworthy that, although most studies on MT attribute cellular protection to enhanced OXPHOS, our metabolomic analysis of ECs subjected to MT (Mito group) versus untreated controls revealed no increase in OXPHOS activity—consistent with the primary reliance of ECs on glycolysis for energy production. Instead, we identified 92 differentially expressed metabolites (Fig. 2G and Fig. S1C and D), with pathway analysis demonstrating significant up-regulation of pentose/glucuronate interconversion (represented by glucuronic acid), glycolysis (represented by pyruvic acid), and adrenergic signaling (represented by epinephrine) (Fig. 2H and J to L) [25–28]. Notably, MT significantly augmented the levels of autophagic metabolites (Fig. 2I), which included decreases in ubiquinone-2 (facilitating electron transport chain function) and genipin (inhibiting UCP2) (Fig. 2M and N). According to previous studies, it was demonstrated that these compounds promote mitophagy activation [29,30]. Furthermore, the decline in S-adenosylmethionine (SAM) levels correlated with the substantial enhancement of autophagy flux (Fig. 2O) [31]. Collectively, these findings demonstrate that MT rescues EC function not through OXPHOS augmentation, but rather by inducing a metabolic shift toward glycolytic predominance and activating mitophagy—potential mechanisms that collectively enhance ROS-damaged ECs viability and functionality.

### MT enhances ECs vasculogenic capacity through mitophagy-mediated clearance of damaged mitochondria

**Fig. 3.**
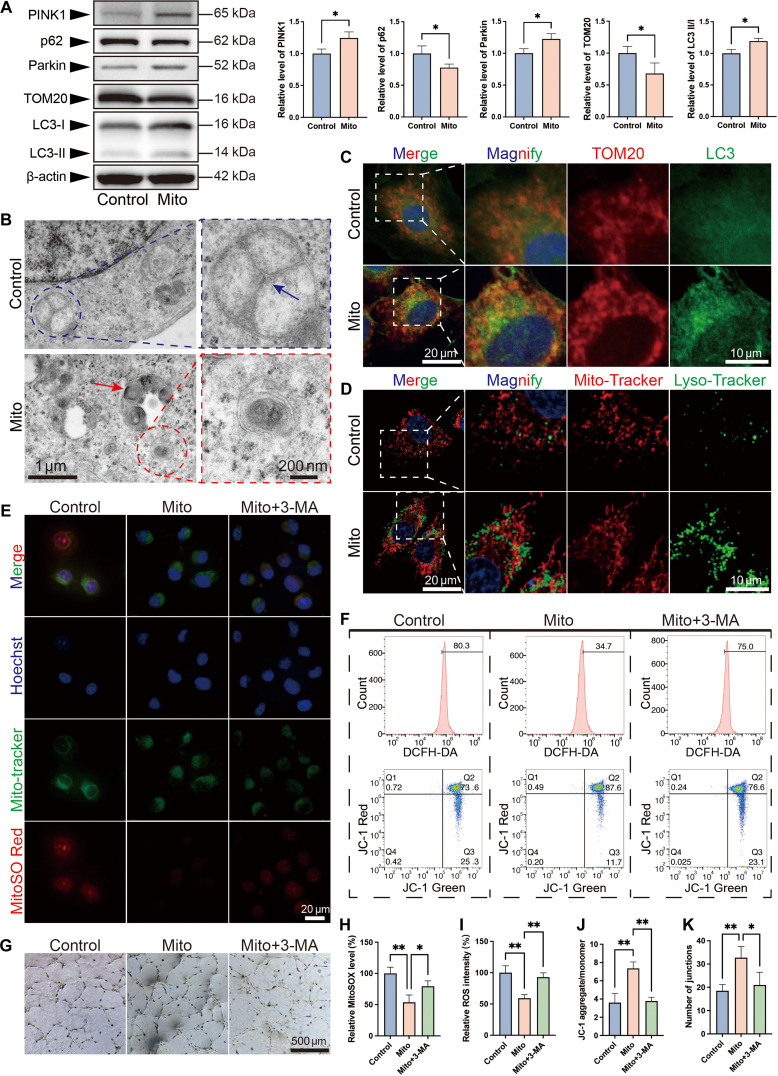
MT enhances ECs’ vasculogenic capacity through clearance of damaged mitochondria. (A) Western blot analysis of autophagy markers (LC3-II/I and p62) and mitophagy markers (PINK1, Parkin, and TOM20) in control ECs versus MT-ECs. β-actin served as loading control. (B) Representative TEM images showing control ECs with swollen mitochondria (blue circle) and disruption and fragmentation of cristae (blue arrow), and MT-ECs containing autolysosomes (red arrow) and mitophagosomes (red circle, double-membrane structures engulfing mitochondria). Scale bars: 1 μm (main image) and 200 nm (magnified view). (C) Confocal microscopy of LC3 (green) and TOM20 (red). Scale bars: 20 μm (main image) and 10 μm (magnified view). (D) Confocal microscopy of lysosome (green) and mitochondria (red). Scale bars: 20 μm (main image) and 10 μm (magnified view). (E) MitoSO Red fluorescence (represent for mtROS) in live ECs under different treatments. Scale bars: 20 μm. (F) Flow cytometry quantification of cellular ROS (DCFH-DA), mitochondrial polarization (JC-1 red/green ratio), and representative pseudocolor plots. (G) Tube formation assays under different treatments. Scale bars: 500 μm. (H to K) Quantitative analyses of (H) mtROS levels, (I) cellular ROS levels, (J) ΔΨm (JC-1 ratio), and (K) angiogenic capacity (number of junctions). Data are presented as mean ± SD, *n* ≥ 3. *P < 0.05, **P < 0.01.

We next investigated whether MT reactivates mitophagy in mitochondria-damaged ECs. Western blot analysis revealed that MT-ECs (Mito group) exhibited increased LC3-II/I ratios and decreased p62 levels compared to controls, indicating enhanced autophagic flux (Fig. 3A). Furthermore, significant up-regulation of PINK1 and Parkin coupled with reduced TOM20 expression provided direct evidence of mitophagy activation (Fig. 3A). Transmission electron microscopy (TEM) revealed significant mitochondrial swelling and cristae disruption in control ECs, which are hallmarks of ROS-induced damage. Conversely, Mito-ECs manifested a substantial presence of autolysosomes and intact double-membrane autophagic vacuoles, which contained mitochondrial remnants (Fig. 3B). The results of immunofluorescence staining were consistent with these findings. MT-ECs displayed increased LC3-mitochondria colocalization (Fig. 3C), with the Pearson’s *R* value (0.35) significantly higher than that of control ECs (0.17) (Fig. S1E). Furthermore, in fluorescence costaining with MitoTracker and Lyso-Tracker, MT-ECs exhibited increased lysosome intensity and enhanced lysosome–mitochondria association compared to control ECs (Fig. 3D). The aforementioned results indicated that MT enhanced both the autophagic flux and mitophagy activation of ECs.

In order to ascertain the extent to which MT-enhanced mitophagy contributes to the clearance of damaged mitochondria and enhanced ECs functionality, we employed a pharmacological approach to inhibit autophagy using 5 mM 3-methyladenine (3-MA). CCK-8 assays demonstrated that autophagy inhibition led to reduced EC viability and significantly attenuated the therapeutic effect of MT, indicating the crucial role of autophagy in mediating the efficacy of MT (Fig. S2A). Similarly, autophagy inhibition resulted in a significant decrease in ATP levels in ECs, suggesting that autophagy itself serves as a critical energy source for these cells (Fig. S2B). Furthermore, while MT treatment significantly elevated pyruvate kinase levels—corroborating enhanced glycolysis—autophagy inhibition did not produce a statistically significant reduction in its activity, suggesting that glycolytic flux is likely regulated through a complex interplay of signaling pathways beyond autophagy alone (Fig. S2C). Tetramethylrhodamine (TMRE) staining revealed that MT augmented mitochondrial membrane potential (ΔΨm) by 32%, yet this enhancement was nearly entirely abrogated following the incorporation of 3-MA (Fig. S2D and E). In a similar manner, MT-mediated reductions in mitochondrial ROS (mtROS) and cellular ROS, along with improved JC-1 aggregation/monomer ratio, were all significantly attenuated upon autophagy inhibition (Fig. 3E, F, and H to J). These findings demonstrate that MT-activated mitophagy selectively eliminates damaged mitochondria, thereby improving overall mitochondrial membrane potential and cellular mitochondrial homeostasis. Subsequently, we obtained consistent results through tube formation assays (Fig. 3G and K and Fig. S2F). Mechanistically, we propose that MT clears dysfunctional mitochondria through mitophagy, which are the primary sources of ROS overproduction under oxidative stress. This process has been shown to terminate pathological ROS emission, restore mitochondrial homeostasis, and consequently potentiate ECs’ vasculogenic capability. In turn, this establishes a self-reinforcing cycle of vascular repair.

### Preparation and characterization of Mito-AVM

The efficacy of conventional MT is fundamentally constrained by its passive dependence on the intrinsic mitochondrial engulfment capacity of recipient cells. This inherent limitation results in poor targeting specificity and low transplantation efficiency. To overcome these limitations, a novel delivery strategy was developed, leveraging ApoVs—a specialized subset of extracellular vesicles that inherit parent cell membrane proteins, including cell-specific ligands, and exhibit surface-exposed phosphatidylserine (“eat-me” signals) [32]. Our previous research demonstrated ApoVs’ dual competence in targeted delivery and cargo loading, which positions ApoVs as ideal biological carriers for precision mitochondrial delivery [23]. However, given the compositional heterogeneity of ApoVs and their potential to induce adverse effects, the uncontrolled content was selectively removed while the functional vesicular membrane was preserved to achieve the desired functionality.

**Fig. 4.**
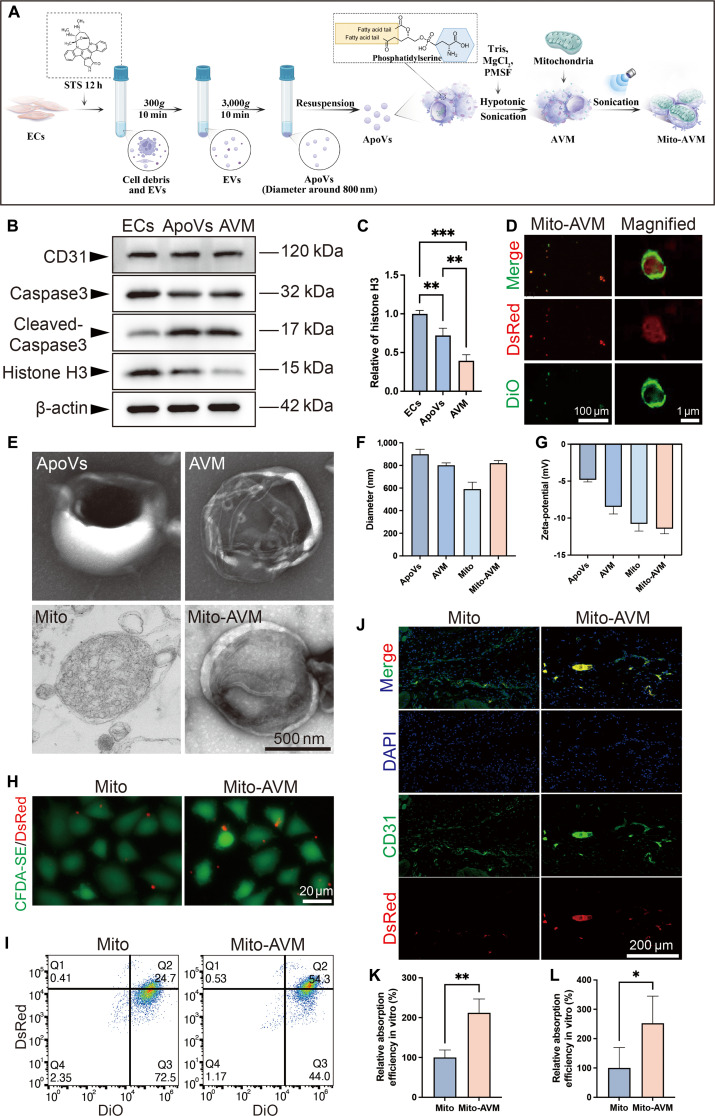
Preparation and characterization of Mito-AVM. (A) Schematic of Mito-AVM fabrication. (B) Western blot analysis of ECs, ApoVs, and AVM: endothelial marker (CD31), apoptosis indicators (caspase-3, cleaved caspase-3), and content marker (histone H3). β-actin loading control. (C) Quantification of histone H3 in ApoVs and AVM. (D) Fluorescence colocalization of DiO-labeled AVM (green) and Mito-DsRed mitochondria (red). Scale bars: 100 μm (main image) and 1 μm (magnified view). (E) TEM micrograph of ApoVs, AVM, mitochondria, and Mito-AVM. Scale bars: 500 nm. (F) Hydrodynamic diameter distributions by dynamic light scattering. (G) Zeta potential measurements. (H) Confocal imaging of ECs after 4 h of incubation with Mito or Mito-AVM. Scale bars: 20 μm. (I) Flow cytometric quantification of mitochondrial uptake. (J) In vivo tracking immunofluorescence images of tissue sections 24 h post-subcutaneous injection in diabetic wounds. Scale bars: 200 μm. (K and L) Quantitative analysis of mitochondrial uptake: (K) in vitro (flow cytometry) and (L) in vivo (fluorescence intensity per CD31^+^ area). Data are presented as mean ± SD, *n* ≥ 3. *P < 0.05, **P < 0.01, ***P < 0.001.

Consequently, a biohybrid delivery system was developed by fusing EC-derived AVM with isolated mitochondria (Fig. 4A). First, we induced ECs apoptosis by staurosporine (STS), extracted ApoVs by differential centrifugation, and further removed its contents by hypotonic treatment for 1 h and sonication for 10 min to obtain AVM. The marker proteins of ApoVs and AVM were verified by Western blot analysis, and it was demonstrated that AVM removed its contents after treatment (represented by histone H3) (Fig. 4B and C). Subsequently, the DiO-stained AVM was coincubated with mitochondria extracted from healthy ECs that were transfected with mito-DsRed in a 2:1 ratio. By means of mild ultrasound and centrifugation, the Mito-AVM was obtained. Fluorescent colocalization indicated that the fusion efficiency approached 95%, and the structure of AVM encapsulating mitochondria was distinctly discernible (Fig. 4D). The TEM analysis of Mito-AVM cross-sections revealed characteristic mitochondrial cristae structures, while localized outer membrane thickening indicative of membrane fusion was observed (Fig. 4E). The physical characterization of Mito-AVM was performed using dynamic light scattering, which yielded average diameters of 820 ± 22.9 nm (Fig. 4F and Fig. S3A). Additionally, the zeta potentials were determined to be −11.4 ± 0.7 mV (Fig. 4G). Oxidative stress testing demonstrated the superior stability of Mito-AVM compared to free mitochondria (Fig. S3B), which may be due to the protective effect of AVM on mitochondria. Theoretically, Mito-AVM leverages the phosphatidylserine present on the AVM surface along with homologous targeting proteins to promote its uptake by ECs via endocytosis. The complex is internalized as an integrated entity and, upon entering the ECs, acts to reactivate the mitochondrial autophagy process. Subsequently, by coculturing free mitochondria and Mito-AVM with ECs for 4 h respectively, it was found that ECs exhibited a significantly higher uptake efficiency of Mito-AVM (Fig. 4H). The results of flow cytometry demonstrated a 110% higher uptake efficiency for Mito-AVM compared to free mitochondria (Fig. 4I to K). Concurrently, the wound model of diabetic rats was established, and the same number of free mitochondria and Mito-AVM were subcutaneously injected into the rats, respectively. After 24 h, it was found that the MT efficiency of Mito-AVM was significantly higher (152%) than that of free mitochondria in vivo (Fig. 4J to L).

### Mito-AVM exhibits enhanced capacity to promote damaged mitochondrial clearance and restore cellular function in mitochondria-damaged ECs

**Fig. 5.**
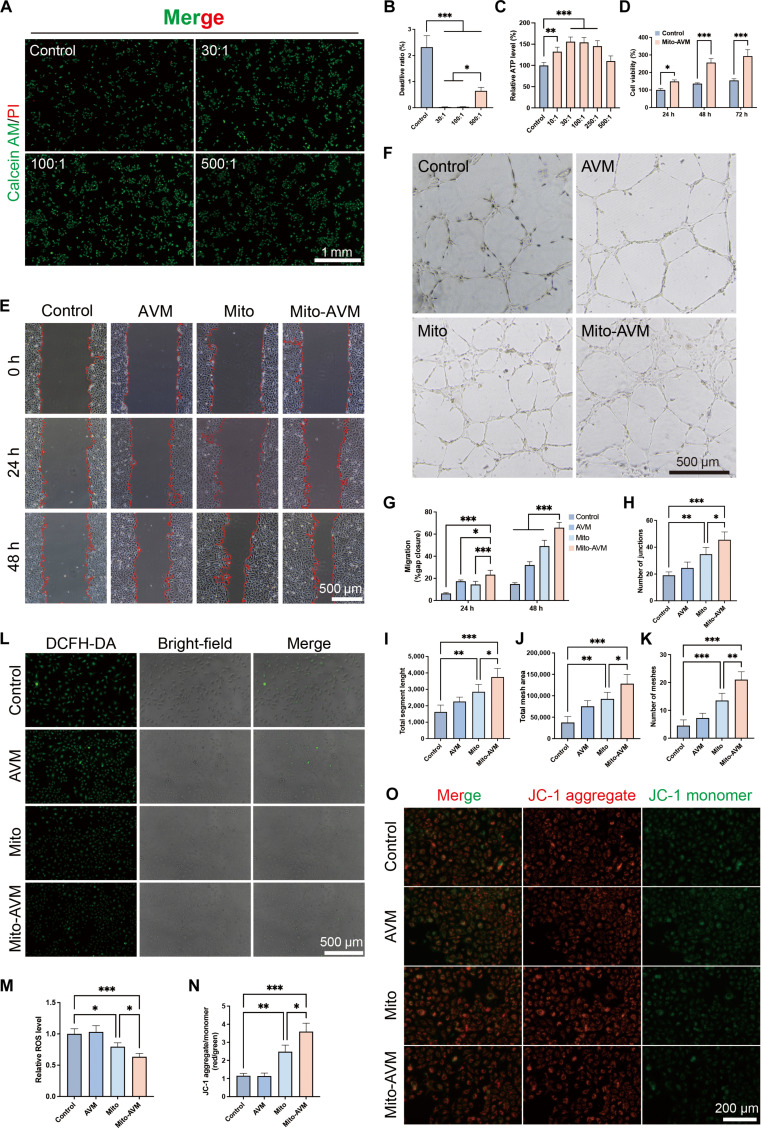
Mito-AVM exhibits enhanced capacity to promote damaged mitochondrial clearance and restore cellular function. (A) Live/dead staining of ECs treated with different Mito-AVM:EC ratios for 24 h. Scale bars: 1 mm. (B) Quantitative dead/live analysis. (C) Relative normalized ATP levels. (D) CCK-8 proliferation kinetics at 100:1 ratio over 72 h. (E) Scratch wound healing assay. Scale bars: 500 μm. (F) Tube formation assay. Scale bars: 500 μm. (G) Quantified gap closure rate. (H to K) Vasculogenic parameters: (H) Vascular junctions, (I) total segment length, (J) meshed area, and (K) mesh number. (L) Cellular ROS detection (DCFH-DA) after 48-h treatments. Scale bars: 500 μm. (M) Quantified ROS intensity. (N) JC-1 aggregate/monomer ratio (ΔΨm indicator). (O) JC-1 fluorescence (red: aggregates; green: monomers). Scale bars: 200 μm. Data are presented as mean ± SD, *n* ≥ 3. *P < 0.05, **P < 0.01, ***P < 0.001.

To determine whether Mito-AVM exhibits a superior therapeutic effect on mitochondria-damaged ECs, rigorous evaluations were conducted. Dose optimization via CCK-8 assay identified 1 μg/ml as the optimal AVM concentration for EC viability (Fig. S4A). At this concentration, subsequent live/dead staining at various MT-AVM number-to-cell number ratios revealed maximal cyto-protection at ratios of 30:1 to 100:1. However, the 500:1 ratio induced cytotoxicity, potentially due to excessive autophagy-triggered apoptosis (Fig. 5A and B). Cellular ATP quantification revealed a dose-dependent ATP level proportional to transplanted mitochondrial load at ratios <100:1, reaching saturation at ratios ≥100:1 (Fig. 5C). The CCK-8 assay also demonstrated that ECs treated with Mito-AVM at the 100:1 ratio exhibited excellent proliferative capacity within 72 h (Fig. 5D). Consequently, we employed the optimized 100:1 ratio for subsequent functional analyses.

Next, we validated EC function and intracellular mitochondrial status in the following groups: control, AVM, Mito, and Mito-AVM. In scratch and tube formation assays (Fig. 5E and F), the Mito-AVM group demonstrated superior cell migration (Fig. 5G) and tube formation abilities (Fig. 5H to K). Additionally, ROS and JC-1 staining revealed that, after 48 h of treatment, the Mito-AVM group exhibited the lowest ROS levels (Fig. 5L and M) and the optimal mitochondrial membrane potential (Fig. 5N and O). Finally, we subcutaneously injected AVM, Mito, and Mito-AVM into the wounds of diabetic rats, respectively. After 48 h, we took samples for Western blot analysis and found that the Mito-AVM group exhibited the strongest mitophagy (Fig. S4B). The aforementioned results collectively indicate that Mito-AVM is more effective than free mitochondria at enhancing the clearance of damaged mitochondria and restoring cellular function in mitochondria-damaged ECs.

### Fabrication, characterization, and functional validation of the Mito-AVM@HPP hydrogel

**Fig. 6.**
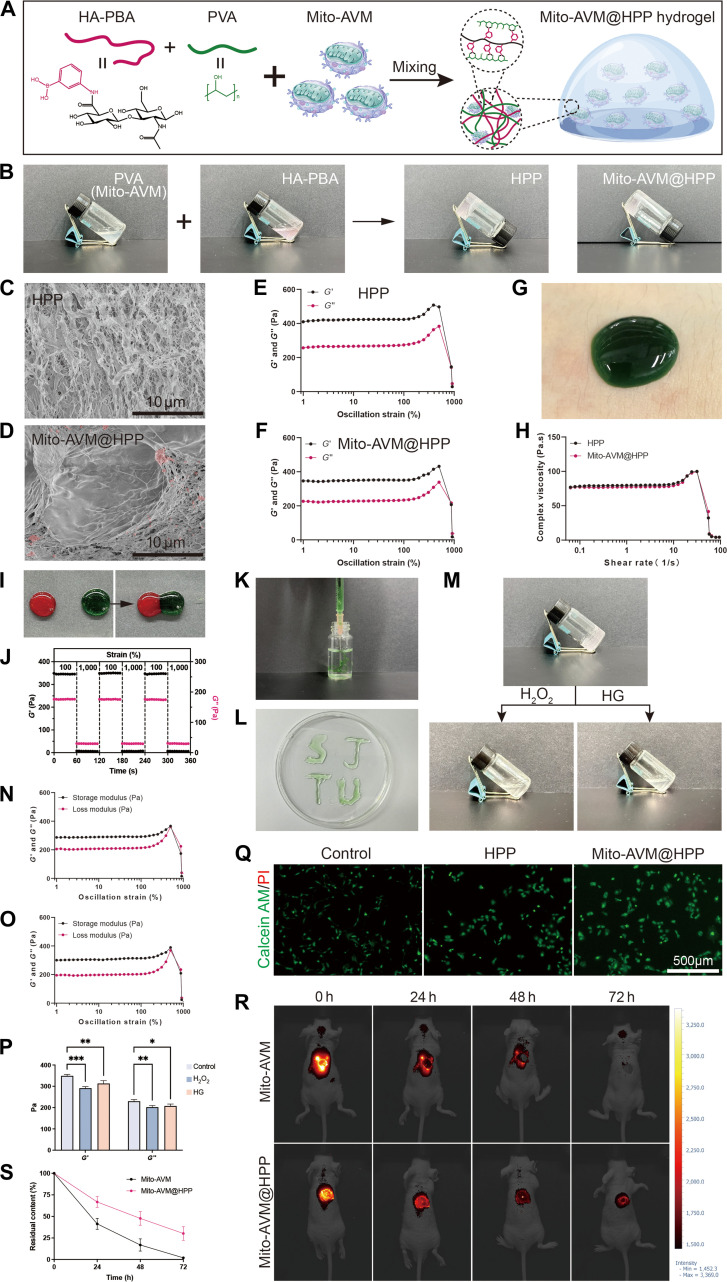
Fabrication, characterization, and functional validation of the Mito-AVM@HPP hydrogel. (A) Schematic of the Mito-AVM@HPP hydrogel fabrication via boronate-diol crosslinking. (B) The gelation process of the HPP and Mito-AVM@HPP hydrogels. (C and D) Morphological observation of the HPP and Mito-AVM@HPP hydrogels via scanning electron microscope (red: clustered Mito-AVM). Scale bars: 10 μm. (E and F) *G*′ and *G*″ of the HPP and Mito-AVM@HPP hydrogels in the strain sweep test. (G) Macroscopic adhesion to human skin. (H) Viscosity test. (I) Autonomous self-healing of segmented hydrogels. (J) Step-strain tests of the Mito-AVM@HPP hydrogel at 37 °C applying 100% strain for 60 s and 1,000% strain for 60 s repeatedly. (K and L) Injectable performance: (K) Extrusion through a 21G needle and (L) precise “SJTU” patterning. (M) Complete degradation in 100 μM H_2_O_2_ and 50 mM glucose after 7 days. (N to P) Moduli reduction after 72 h of exposure to 100 μM H_2_O_2_ and 50 mM glucose. (Q) Live/dead staining of ECs after 24 h of hydrogel contact. Scale bars: 500 μm. (R) Bioluminescence tracking of Mito-AVM retention. (S) Quantified wound fluorescence intensity. Data are presented as mean ± SD, *n* ≥ 3. *P < 0.05, **P < 0.01, ***P < 0.001.

To address the rapid clearance of Mito-AVM in diabetic wounds, we engineered the Mito-AVM@HPP hydrogel enabling spatiotemporal release through dynamic boronic ester bond crosslinking between HA-PBA and PVA (Fig. 6A). First, HA was functionalized with PBA through amide-bond conjugation. Successful HA-PBA synthesis was confirmed by ^1^H-NMR (Fig. S5A). A uniform mixture was prepared by combining 20 mg/ml HA-PBA and 60 mg/ml PVA at a 1:1 volume ratio, which underwent rapid crosslinking and gelation via boronic ester bond formation. At this stage, the HPP hydrogel was successfully synthesized, and as demonstrated, it can firmly adhere to a glass surface and resist gravitational forces (Fig. 6B). The optimal concentration of Mito-AVM encapsulated within the HPP hydrogel was determined to be 5 μg/ml via CCK-8 assay (Fig. S5B). At this concentration, the Mito-AVM@HPP hydrogel demonstrated the most pronounced effect in promoting ECs viability in vitro. Consequently, this concentration was employed in all subsequent experiments. Cryo-SEM revealed the presence of porous networks and the Mito-AVM distribution (Fig. 6C and D).

The rheological properties of the HPP and Mito-AVM@HPP hydrogels were first characterized by strain sweep tests, which confirmed that both hydrogels exhibited gel-like viscoelastic behavior within the 1% to 100% strain range. However, as the strain increased to 1,000%, storage modulus (*G*′) and loss modulus (*G*″) decreased, and inversion occurred (*G*″ > *G*′), demonstrating the marked shear-thinning behavior of the hydrogels (Fig. 6E and F). Viscosity tests and skin adhesion experiments demonstrated that the hydrogels possess favorable adhesion properties, rendering them well-suited for dermal applications (Fig. 6G and H).

Next, we evaluated both the self-healing properties and the injectability of the hydrogels. In the context of wound healing applications, the injectability and self-healing properties of hydrogels play a crucial role in promoting the healing process. The injectability enables minimally invasive administration, allowing the hydrogel to adapt seamlessly to irregular wound contours, thereby ensuring complete coverage and intimate tissue contact. Furthermore, the rapid self-healing capacity guarantees structural integrity within the dynamic wound environment. Under continuous mechanical stress induced by body movement, the hydrogel autonomously repairs damage, maintaining an uninterrupted physical barrier against pathogenic invasion and preventing structural failure [33]. After being cut in half with a scalpel, the 2 hydrogel segments exhibited interfacial blurring and autonomously coalesced into an integral hydrogel structure, demonstrating its intrinsic self-healing capability (Fig. 6I). Concurrently, step-strain testing (strain = 100% to 1,000%) over 3 consecutive cycles revealed rapid network fracture at 1,000% strain, evidenced by precipitous declines in *G*′ and *G*″. Subsequent strain reduction triggered immediate modulus recovery, demonstrating the self-healing capability of the hydrogels (Fig. 6J). The hydrogels’ injectability was demonstrated by its smooth extrusion through 21G needles (Movie S1) and its ability to deposit “SJTU” patterns (acronym for Shanghai Jiao Tong University) while preserving structural integrity (Fig. 6K and L).

Previous studies have reported that boronic ester bonds are sensitive to ROS and glucose, and therefore, hydrogel networks may be disrupted when used on diabetic wounds [34]. We investigated whether Mito-AVM@HPP hydrogel could be degraded by ROS and glucose to release Mito-AVM. To confirm the ROS/glucose dual responsiveness degradation of the hydrogels, we used solutions of 100 μM H_₂_O_₂_ and 50 μM glucose to treat hydrogels respectively. After 7 days of treatment, it was observed that the hydrogel had almost completely self-degraded into liquid (Fig. 6M). Concurrently, strain sweep tests revealed marked reductions in both *G*′ and *G*″ for the hydrogels exposed to 100 μM H_₂_O_₂_ and 50 mM glucose for 72 h compared with untreated controls (Fig. 6N to P). These findings demonstrate favorable ROS/glucose dual-responsive biodegradation kinetics of the hydrogels.

We assessed the therapeutic efficacy of the hydrogels in practical application. A biocompatibility assessment via live/dead staining revealed that Mito-AVM@HPP not only exhibited excellent cytocompatibility, but significantly enhanced ECs proliferation after 24 h of coculture (Fig. 6Q). In diabetic murine wound models, in vivo tracking of DiD-labeled Mito-AVM revealed that conventional intradermal administration suffered rapid clearance (remaining 41.4% ± 6.3% at 24 h; 16.8% ± 7.2% at 48 h; 2.0% ± 1.4% at 72 h), whereas topical application of Mito-AVM@HPP enabled sustained release kinetics (remaining 66.8% ± 6.2% at 24 h; 47.7% ± 7.9% at 48 h; 30.2% ± 7.9% at 72 h) (Fig. 6R and S). This validates its capacity to overcome clearance barriers through spatiotemporally controlled delivery.

### Mito-AVM@HPP hydrogels accelerate diabetic wound healing

**Fig. 7.**
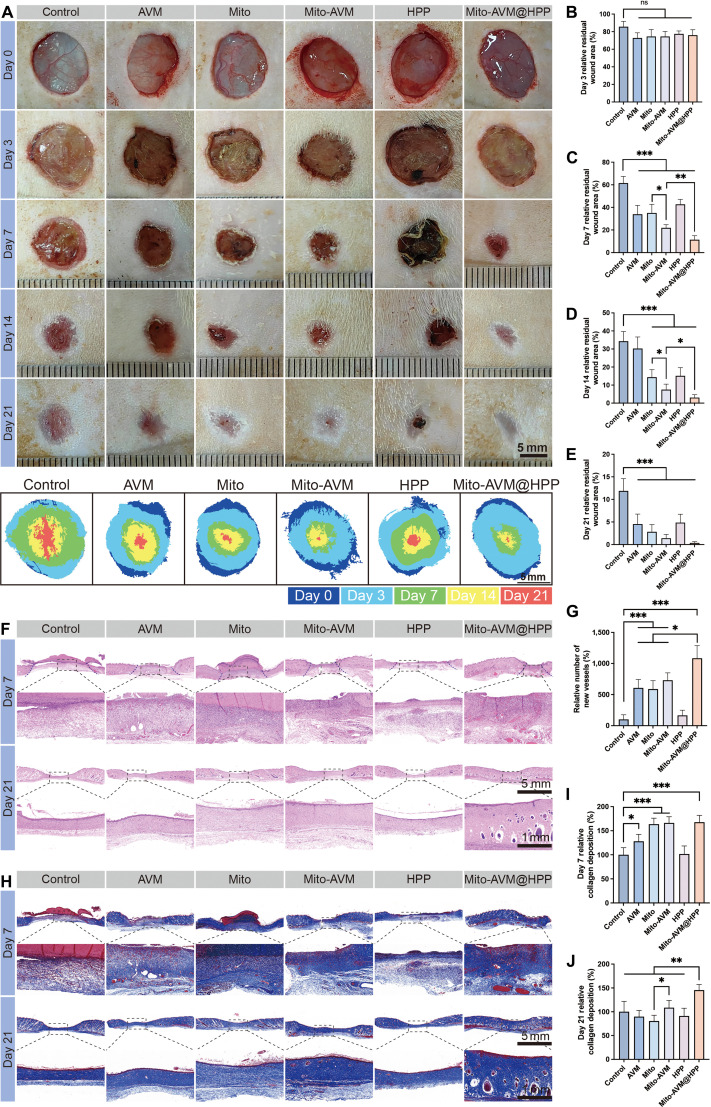
The effects of each treatment on diabetic wound healing. (A) Representative wound images from Control, AVM, MT, Mito-AVM, HPP, and Mito-AVM@HPP groups on days 0, 3, 7, 14, and 21 post-treatments. Scale bars: 5 mm. (B to E) Quantitative analysis of wound closure percentage on days 3, 7, 14, and 21. (F) Representative H&E staining images. Scale bars: 5 mm (main image) and 1 mm (magnified view). (G) Comparative analysis of neovascularization. (H) Representative Masson’s trichrome-staining images. Scale bars: 5 mm (main image) and 1 mm (magnified view). (I to J) Quantification analysis of relative collagen deposition on day 7 and day 21. Data are presented as mean ± SD, *n* ≥ 3. *P < 0.05, **P < 0.01, ***P < 0.001.

Using an established STZ-induced diabetic rat model [35], we observed residual wound area across 6 experimental groups (Control, AVM, Mito, Mito-AVM, HPP, and Mito-AVM@HPP) on days 0, 3, 7, 14, and 21 (Fig. 7A). The Mito-AVM@HPP hydrogel treatment demonstrated superior therapeutic efficacy on days 7, 14, and 21. Quantitative image analysis revealed a therapeutic hierarchy on days 7 and 14 that group Mito-AVM@HPP > Mito-AVM > AVM or Mito or HPP > Control (Fig. 7B to E). These results conclusively establish that the Mito-AVM@HPP hydrogel is the most effective at accelerating diabetic wound healing.

Additionally, rats were euthanized at 2 stages of wound healing: early (day 7) and late (day 21). Histological staining was performed, including hematoxylin and eosin (H&E) staining and Masson’s trichrome staining. H&E staining analysis revealed that, on day 7 post-treatment, the Control group had the largest wound gap of the 6 experimental groups, while the Mito-AVM@HPP group had the smallest wound length (Fig. 7F). Quantitative assessment of neovascularization revealed significantly higher numbers of new blood vessels in the AVM, Mito, Mito-AVM, and Mito-AVM@HPP groups than in the Control group (Fig. 7G). The Mito-AVM@HPP group demonstrated significantly greater neovascularization than the AVM, Mito, and Mito-AVM groups, indicating the greatest improvement in the prognosis of diabetic wound healing. By day 21, 4 treatment groups (Mito, Mito-AVM, and Mito-AVM@HPP) had comparable levels of complete wound closure; however, the Mito-AVM@HPP group had superior tissue maturation. Masson’s trichrome staining (Fig. 7H) revealed increased collagen deposition (blue staining) in the Mito, Mito-AVM, and Mito-AVM@HPP groups compared to the Control group on day 7 (Fig. 7I). On day 21, the Mito-AVM@HPP group exhibited significantly greater collagen accumulation than the other groups (Fig. 7J).

**Fig. 8.**
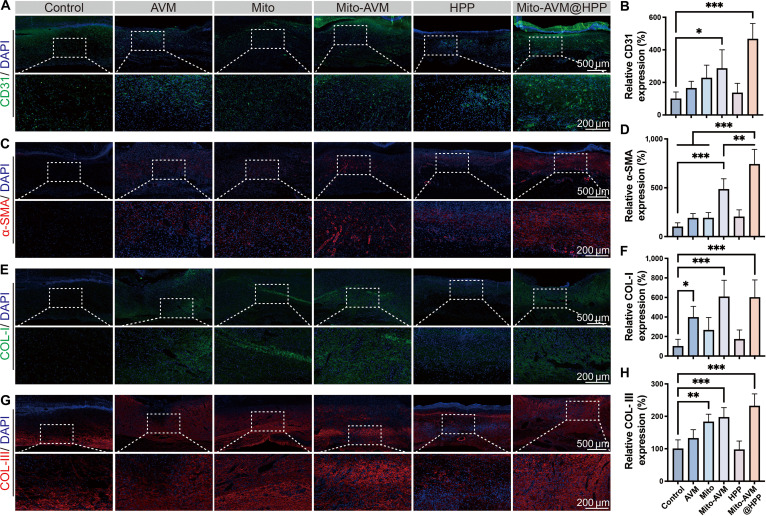
The effects of each treatment on vascularization, vascular maturation, and collagen deposition on day 7. (A) Representative CD31 images. Scale bars: 500 μm (main image) and 200 μm (magnified view). (B) Relative CD31 expression. (C) Representative α-SMA images. Scale bars: 500 μm (main image) and 200 μm (magnified view). (D) Relative α-SMA expression. (E) Representative COL-I images. Scale bars: 500 μm (main image) and 200 μm (magnified view). (F) Relative COL-I expression. (G) Representative COL-III images. Scale bars: 500 μm (main image) and 200 μm (magnified view). (H) Relative COL-III expression. Data are presented as mean ± SD, *n* ≥ 3. *P < 0.05, **P < 0.01, ***P < 0.001.

To evaluate the therapeutic effects on wound healing systematically, immunofluorescence staining was performed on days 7 and 21 for the EC marker CD31, the pericyte marker α-smooth muscle actin (α-SMA), and collagen types I/III (COL-I/III). Immunofluorescence staining of day 7 wound tissues exhibited varying levels of therapeutic effect among the treatment groups (Fig. 8). Quantitative analysis revealed the following: (a) Vascularization: Control wounds exhibited minimal CD31 expression. In contrast, the MT groups (Mito, Mito-AVM, and Mito-AVM@HPP) showed progressive improvement. Among these groups, group Mito-AVM@HPP demonstrated the highest CD31 expression (Fig. 8A and B). This indicates enhanced ECs proliferation. α-SMA immunofluorescence showed a similar trend, indicating enhanced synchronous vascular maturation (Fig. 8C and D). (b) Collagen deposition: The Mito-AVM@HPP hydrogel significantly increased the expression of both COL-I and COL-III compared to the Control group, demonstrating superior extracellular matrix remodeling capacity (Fig. 8E to H).

Immunofluorescence analysis of late-stage wound healing on day 21 revealed similar therapeutic trends and demonstrated critical phase-specific collagen remodeling (Fig. S6). Notably, quantitative analysis of collagen isoform transitions demonstrated that Mito-AVM@HPP hydrogel treatment optimally regulated the temporal progression of extracellular matrix remodeling over time. While the Control group, the AVM group, the Mito group, and the Mito-AVM group showed the expected shift from COL-III-dominated provisional matrix formation on day 7 to COL-I-mediated stabilization on day 21, the Mito-AVM@HPP group achieved significantly superior collagen maturation evidenced by the highest COL-I/COL-III ratio among all treatment groups on day 21. This reflects both enhanced COL-I deposition and appropriate COL-III resolution. These findings confirm that the Mito-AVM@HPP hydrogel accelerates diabetic wound healing by promoting endothelial proliferation and vascular maturation, as well as enhancing collagen synthesis and deposition, with greater efficacy than either Mito or Mito-AVM alone.

## Conclusion

MT has emerged as a promising organelle-based therapy for mitochondrial-related diseases, yet its underlying therapeutic mechanism requires further clarification. Here, we specifically examine the mechanism of MT in mitochondria-damaged ECs. In contrast to highly metabolic cell types such as cardiomyocytes and alveolar epithelial cells, which often incorporate transplanted mitochondria into their existing network to support OXPHOS [13,36–38], ECs exhibit relatively low mitochondrial abundance and primarily serve as signaling regulators rather than bioenergetic hubs [39]. Consistent with this biological context, we observed that MT did not enhance overall mitochondrial content but instead activated PINK1-Parkin-mediated mitophagy. We propose that under conditions of chronic oxidative injury, where dysfunctional mitochondria accumulate, MT preferentially triggers a quality-control response that promotes the clearance of damaged mitochondria. This clearance process subsequently facilitates functional recovery through cytoprotective downstream pathways rather than through bioenergetic augmentation [18].

Beyond mitophagy restoration, we observed enhanced glycolysis in MT-treated ECs. Although this may be a downstream change induced by mitochondrial autophagy, this phenomenon should be interpreted with caution. ECs typically rely on glycolysis for ~80% of their ATP production (Warburg-like effect) [40–42], and glycolysis critically regulates EC migration and vasculogenesis [41]. Although studies have shown that the mtDNA of transplanted mitochondrial is completely cleared by recipient ECs over time [18], further investigation is warranted to determine whether other components remain undegraded in the short term and directly regulate physiological processes in cells, such as glycolysis.

To overcome the challenges of MT in practical applications, we used a combination of bioengineering and biomaterials to optimize MT at cellular, tissue, and spatiotemporal levels. At the cellular level, we used bioengineered mitochondrial modifications. While previous mitochondrial delivery strategies have employed cationic liposomes or cell membrane coatings to provide protection against extracellular degradation [43,44], these systems exhibit distinct functional limitations. Cationic liposomes bind to mitochondria through electrostatic interactions and facilitate delivery to cells through membrane fusion. However, they lack intrinsic targeting specificity and exhibit suboptimal delivery efficiency and potential biological toxicity. Cell membrane-based coatings offer inherent homing capabilities but are limited by their large size relative to mitochondria, which physically impairs delivery performance. In contrast, AVM represents an optimally designed carrier that integrates the advantages of both systems while addressing their individual shortcomings. AVM exhibits excellent size compatibility with mitochondria, enabling highly efficient encapsulation. Furthermore, AVM presents natural “eat-me” signals, which promote specific recognition and active uptake by ECs through efferocytosis-like mechanisms. In this study, AVM possesses mitochondrial protective capabilities and exhibits approximately 150% higher targeting ability and transplantation efficiency than free mitochondria in vivo, establishing it as a highly promising platform for MT therapy.

To optimize tissue and spatiotemporal levels, we designed a hydrogel (HPP) tailored to the unique microenvironment of diabetic wounds. Hydrogels are ideal for wound healing; they close the wound while absorbing secreted tissue fluid, thereby facilitating wound closure [45,46]. The HPP hydrogel has excellent physical properties and can be easily injected into the wound using a syringe. Furthermore, the hydrogel undergoes responsive degradation and releases Mito-AVM, which exerts therapeutic effects in the high-glucose, high-ROS environment of diabetic wounds. These properties make the hydrogel superior and conducive to clinical translation.

In summary, we elucidated the mechanism that MT clears damaged mitochondria of ECs by reactivating mitophagy. Additionally, this work advances the treatment of diabetic chronic wounds by integrating organelle-level bioengineering with biomaterial design. It may hopefully provide a versatile framework for MT therapy in diseases characterized by mitochondrial dysfunction.

## Materials and Methods

### Cell culture

Human umbilical vein endothelial cells (HUVECs; Anwei Biotechnology, Shanghai, China), hereinafter referred to as ECs, were cultured at 37 °C using Endothelial Cell Medium (ScienCell, 1001). For all experiments, ECs between passages 6 and 12 were utilized. To simulate mitochondria damage, 100 μM H_₂_O_₂_ was used to treat ECs for 24 h prior to experiments.

### Labeling mitochondria with mitoDsRed

Mitochondria labeled with DsRed were prepared by transfecting ECs with the pLV-CMV-MCS-mitoDsRed-Puro plasmid (MiaoLing plasmid, China). Following transfection, efficient visualization of mitochondria was achieved under a fluorescence microscope.

### Mitochondrial isolation and MT

The Cell Mitochondria Isolation Kit (Beyotime, C3601, China) was used to isolate mitochondria from donor cells. The procedure was as follows: the cells were digested with trypsin from the culture dish, washed twice with cold phosphate-buffered saline (PBS) and gently resuspended in mitochondrial isolation reagent supplemented with phenylmethanesulfonylfluoride (PMSF). After incubating at 4 °C for 20 min, the cell suspension was homogenized 40 times. Then, the homogenate was centrifuged at 1,000*g* at 4 °C for 10 min, and the supernatant was taken, and then centrifuged at 3,500*g* at 4 °C for 15 min to obtain isolated mitochondria. The mitochondrial pellet obtained from the isolation procedure was stained with MitoTracker, allowing clear visualization of the morphology of the free mitochondria. Concurrently, JC-1 staining was performed to validate their functional activity.

For MT, isolated mitochondria (or Mito-AVM/Mito-AVM@HPP) were added to the culture medium in appropriate amounts and incubated with ECs in a conventional culture environment. Twelve hours after MT, ECs were washed 3 times with PBS, and then cultured with new ECM.

### Cell viability assessment by live/dead staining

Cell viability was evaluated at 24 h post-MT by the Live/Dead Staining Kit (Sigma-Aldrich, 04511). The cells were incubated with the diluted dye at 37 °C for 30 min, and then observed with a fluorescence microscope and photographed. Quantitative assessment was performed using ImageJ software (NIH, USA) to measure fluorescence intensity and positive staining area for each experimental group.

### CCK-8 assay

Add the CCK-8 reagent (Beyotime, C0038) to a 96-well plate containing cells, incubate for 30 to 45 min, and measure the absorbance at 450 nm by a microplate reader (Thermo Scientific, Varioskan Flash 3001).

### Migration assay

The migratory capacity of ECs was evaluated using a standard scratch wound assay. In a 6-well plate, after ECs reached confluence (80%–90%), use a 1,000-μl pipette tip to draw a line along the center of the plate, followed by immediate treatment such as MT. Wound healing was photographed and observed at 0-, 24-, and 48-h post-treatment. Using ImageJ software (NIH, USA), the remaining wound area/initial wound area was compared to quantitatively analyze the migration ability of ECs at each point in time.

### Angiogenesis assay

The angiogenic capacity of ECs was evaluated using the following experimental protocol. The specific steps are as follows: Matrigel (Corning, 356234) was thawed overnight at 4 °C, and prechilled 24-well culture plates were prepared. Add 50 μl of Matrigel to each well and incubate at 37 °C in a cell culture incubator for 60 min. Cells with different treatments were digested with trypsin and suspended in complete culture medium with 5 × 10^5^ cells/ml. Carefully add 200 μl of cell suspension to each Matrigel-coated well. Remove the plate from the incubator after 6 h of incubation and assess tubule formation using a phase-contrast microscope. Network structures were quantified by measuring number of junctions, total segment length, total mesh area, and number of meshes using ImageJ software (NIH, USA) with the Angiogenesis Analyzer plugin.

### Assessment of normalized cellular ATP levels

Cellular ATP levels were detected by the ATP Detection Kit (Beyotime, S0026). Briefly, cells were treated with ATP lysis buffer for 30 min, then the detection solution is added, and the luminescence of each well was measured by a microplate reader. ATP levels were normalized accordingly to protein concentrations determined by Bradford assay.

### Metabolomic profiling of MT effects

To evaluate the effect of MT in mitochondria-damaged ECs, cells were first subjected to oxidative injury by 24 h of treatment with 100 μM H_₂_O_₂_, followed by MT at a 100:1 ratio. After 72 h, a comprehensive metabolomics analysis was performed on the 2 cell groups using integrated liquid chromatography–mass spectrometry and gas chromatography–mass spectrometry platforms, which were provided by Shanghai Luming Biotechnology Co., Ltd. (Shanghai, China). The specific analysis process and data statistical methods were the same as in our previous study [33].

### Western blot analysis

Wash the ECs in a 10-cm dish 3 times with PBS, digest the ECs with trypsin, centrifuge the digested ECs, wash them 3 times with PBS again, and then lyse them with RIPA lysis buffer (Thermo Fisher Scientific) containing both phosphatase and protease inhibitor (Roche Applied Science). The lysate was then incubated on ice for 20 to 30 min and subsequently centrifuged at 14,000*g* for 15 min at 4 °C. The protein samples were then diluted with 4× Laemmli buffer containing 10% β-mercaptoethanol (Bio-Rad, 1610747) and denatured at 95 °C for 5 min. Load 25 μg of protein per lane onto the gel, separate by sodium dodecyl sulfate polyacrylamide gel electrophoresis (SDS-PAGE), and transfer to a polyvinylidene fluoride (PVDF) membrane (Sigma-Aldrich). ThePVDF membrane was blocked with rapid blocking solution (Beyotime, P0240) at room temperature for 10 min. Coincubate the PVDF membrane with the primary antibody. After 3 10-min washes with TBST, the PVDF membrane was incubated with the appropriate secondary antibody at room temperature for 1 h, followed by 3 additional TBST washes. Protein bands were visualized using ECL solution (Proteintech) and imaged.

Primary antibodies included the following: Anti-LC3 (Proteintech, 14600-1-AP, 1:1,000), Anti-P62/SQSTM1 (Proteintech, 18420-1-AP, 1:1,000), Anti-PINK1 (Proteintech, 23274-1-AP, 1:1,000), Anti-Parkin (Proteintech, 14060-1-Ap, 1:1,000), Anti-TOM20 (Proteintech, 11802-1-AP, 1:1,000), Anti-β-actin (Proteintech, 20536-1-AP, 1:2,000), Anti-CD31 (Proteintech, 66065-2-Ig, USA, 1:5,000), Anti-Caspase3 (Affinity, AF6311, 1:1,000), Anti-Cleaved-Caspase3 (CST, 9664T, 1:1,000), and Anti-histone H3 (Abcam, Ab201456, 1:2,000).

### Live-cell imaging of mitochondria and lysosomes

Following MT, ECs in glass-bottom dishes (NEST, 801002) were incubated for 48 h. Live cells were then stained with 200 nM MitoTracker Red CMXRos (Beyotime, C1035; 20 min, 37 °C). After washing by PBS, cells were then stained with 500 nM LysoTracker Green DND-26 (Thermo Fisher, L7526; 30 min, 37 °C) and given final PBS washes. Confocal imaging (Zeiss LSM 800, 40×, sequential mode, 488 nm/587 nm lasers) was conducted in complete growth medium with minimized light exposure.

### Assessment of mtROS

Mitochondrial superoxide levels were assessed using MitoSO Red (Beyotime, S0061S). Cells in 6-well plates were treated for 48 h under experimental conditions. Following 2 PBS washes, cells were incubated with 2.5 μM MitoSO Red working solution for 30 min at 37 °C. After removal of the staining solution and 2 additional PBS washes, mitochondrial superoxide production was immediately visualized and quantified using fluorescence microscopy.

### Assessment of cellular ROS

ECs were directly stained with 10 μM DCFH-DA (ROS Detection Kit, Beyotime, S0033S) at 37 °C for 15 min, washed 3 times with PBS, and then immediately analyzed for cellular ROS levels by fluorescence microscopy and flow cytometry (FITC channel).

### JC-1 staining

JC-1 staining (Beyotime, C2006) assessed mitochondrial membrane potential. After incubating cells with JC-1 staining solution for 20 min at 37 °C and 2 washes (JC-1 Dilution Solution), depolarization was quantified via the red/green fluorescence ratio using microscopy and flow cytometry (FITC/PE channels on trypsinized cells).

### TMRE staining

Following the manufacturer’s protocol (TMRE kit, Beyotime, C2001S), cells underwent TMRE staining in complete medium (30 min, 37 °C, light-protected). After 3 prewarmed PBS washes, it was observed immediately via fluorescence microscopy.

### Immunofluorescence staining

Following fixation by 4% paraformaldehyde for 15 min, permeabilization by 0.1% Triton X-100/PBS for 10 min, and blocking by 5% normal serum for 1 h, coincubate the primary antibodies with glass-grown cells overnight at 4 °C. Remove the primary antibodies, wash 3 times with PBS, and then incubate the samples with different secondary antibodies at room temperature for 1 h. Then, the samples were washed again 3 times with PBS, sealed with anti-fading medium, and stored at 4 °C away from light.

### Isolation and characterization of the Mito-AVM

ECs cultured under standard conditions (37 °C, 5% CO_₂_) were treated with 5 μM STS (Sigma-Aldrich) for 12 h to produce ApoVs, confirmed by characteristic morphological changes (nuclear membrane rupture and membrane blebbing) via optical microscopy. After trypsinization and centrifugation (300*g*, 10 min), the supernatant was further centrifuged (3,000*g*, 10 min) to isolate ApoVs. Isolated ApoVs were stored in PBS at −80 °C. AVM generation followed established methods [23]: ApoVs were resuspended in hypotonic lysis buffer (10 mM Tris, pH 7.4, 10 mM MgCl_₂_, and 1 mM PMSF), incubated at 4 °C for 1 h, sonicated (10 min; KQ2200E ultrasonic cleaner), then purified by centrifugation (10,000*g*, 10 min) and triple PBS wash. AVM characterization included protein concentration by bicinchoninic acid assay (abs9232 kit, Absin), Size/zeta potential by DLS (Zetasizer Nano ZSE, Malvern), and Identity/purity by Western blot for CD31, caspase-3, cleaved caspase-3, and histone H3.

Mito-AVM was prepared by mixing AVM with free mitochondria at a protein concentration ratio of 2:1. The mixed solution was sonicated gently and intermittently for 5 min. The sample was centrifuged at 3,500*g* for 15 min at 4 °C, the supernatant was removed and washed 3 times with cold PBS, and the particle size and zeta potential were determined with DLS. Prior to fusion, AVM was labeled with DiO, and mitochondria were labeled with DsRed, enabling the specific structure of Mito-AVM to be observed by confocal microscopy.

### Preparation of the Mito-AVM@HPP hydrogel

HA-PBA conjugation followed established protocol [47], with successful conjugation confirmed by ^1^H NMR in D_₂_O. HA-PBA (20 mg/ml) and PVA (Aladdin, CAS:9002-89-5; 60 mg/ml) were separately dissolved in deionized water containing Mito-AVM. Equal volumes of both solutions were vortex-mixed (20 s) to initiate crosslinking, yielding the Mito-AVM@HPP hydrogel.

### Characterization of the Mito-AVM@HPP hydrogel

The microstructure of the hydrogels was obtained by photographing its cross-section with scanning electron microscopy (Hitachi SU3500) after freeze-drying.

A rotational rheometer featuring 20-mm parallel plate geometry was employed for rheological testing. For each test, 400 μl of either HPP or Mito-AVM@HPP hydrogel was loaded onto the temperature-controlled stage maintained at 37 °C. The analysis included the following: Oscillatory Strain Sweep: frequency: 10 rad/s; strain sequence: 100% strain for 60 s and 1,000% strain for 60 s for 3 rounds. Viscosity Profile: Shear rate was systematically increased from 0 to 1,000 s^−1^ while monitoring viscosity changes to evaluate the hydrogel’s shear-thinning behavior and structural recovery properties.

### Diabetic rat wound healing model

Female Sprague Dawley rats (180 to 220 g) received intraperitoneal STZ (50 mg/kg) to induce diabetes, confirmed by tail vein glucose ≥16.7 mmol/l after 1 week. Diabetic rats were randomized to 6 groups (*n* = 4): control, AVM, Mito, Mito-AVM, HPP, and Mito-AVM@HPP. Under sodium pentobarbital anesthesia, dorsal hair removal preceded creation of 4 full-thickness circular wounds (Ø = 10 mm) using a sterile biopsy punch. Wound healing was tracked via standardized digital photography (days 0, 3, 7, 14, and 21), with closure rates quantified as percentage area reduction (vs. day 0) using ImageJ.

### Optical in vivo imaging

Following successful establishment of the diabetic wound model, DiD-labeled Mito-AVMs were administered via subcutaneous injection around the wound periphery, while DiD-labeled Mito-AVM@HPP hydrogel was applied topically to the wound surface. Fluorescence intensity was longitudinally monitored on days 1, 2, and 3 post-administrations using an IVIS Spectrum imaging system (Xenogen, USA) with standardized acquisition parameters (excitation/emission: 640/670 nm). Quantitative analysis of signal intensity (photons/s/cm^2^/sr) within predefined regions of interest was performed using Living Image software to compare tissue retention kinetics between the 2 delivery approaches.

### Histological and immunofluorescence analysis of wound tissues

On days 7 and 21, wound tissues were collected post-injury and underwent fixation in 4% paraformaldehyde (48 h), followed by paraffin embedding and sectioning (7 μm thickness). Histological assessment employed H&E staining to evaluate vascular structures (identified by red blood cell-filled lumens) and Masson’s trichrome to visualize collagen deposition. For immunofluorescence, deparaffinized sections underwent antigen retrieval with either tris-EDTA or citrate buffer, followed by serum blocking (5% to 10%) and overnight incubation at 4 °C with primary antibodies against CD31 (Abcam ab182981, 1:400), α-SMA (HUABIO ET1607-53, 1:1,000), COL-I (HUABIO HA722517, 1:200), or COL-III (HUABIO HA720050, 1:200). After fluorophore-conjugated secondary antibody incubation (1:200) and 4′,6-diamidino-2′-phenylindole (DAPI) counterstaining, fluorescence signals were quantified using ImageJ.

### Statistical analysis

No data exclusion criteria were applied during analysis. Quantitative data were expressed as mean ± SD. Intergroup comparisons used unpaired 2-tailed *t* tests (pairwise) or one-way analysis of variance, following Tukey’s test (multigroup; GraphPad Prism v10.3.1). Statistical significance thresholds were set at *P < 0.05, **P < 0.01, ***P < 0.001.

## Data Availability

The data supporting the results of this study can be obtained from the corresponding author upon reasonable request.
