# Supplementary material for: Apoptotic Vesicle Membrane-Mediated Targeted Endothelial Mitochondrial Transplantation-Clearance Therapy for Diabetic Wound Healing
Source: Research (Wash D C). 2026 Jan 16;9:1042. doi: 10.34133/research.1042 (PMC13123279; doi:10.34133/research.1042)
Supplement: Supplementary 1 — Figs. S1 to S6 Movie S1 [file research.1042.f1.zip › Supplementary Figures.docx]

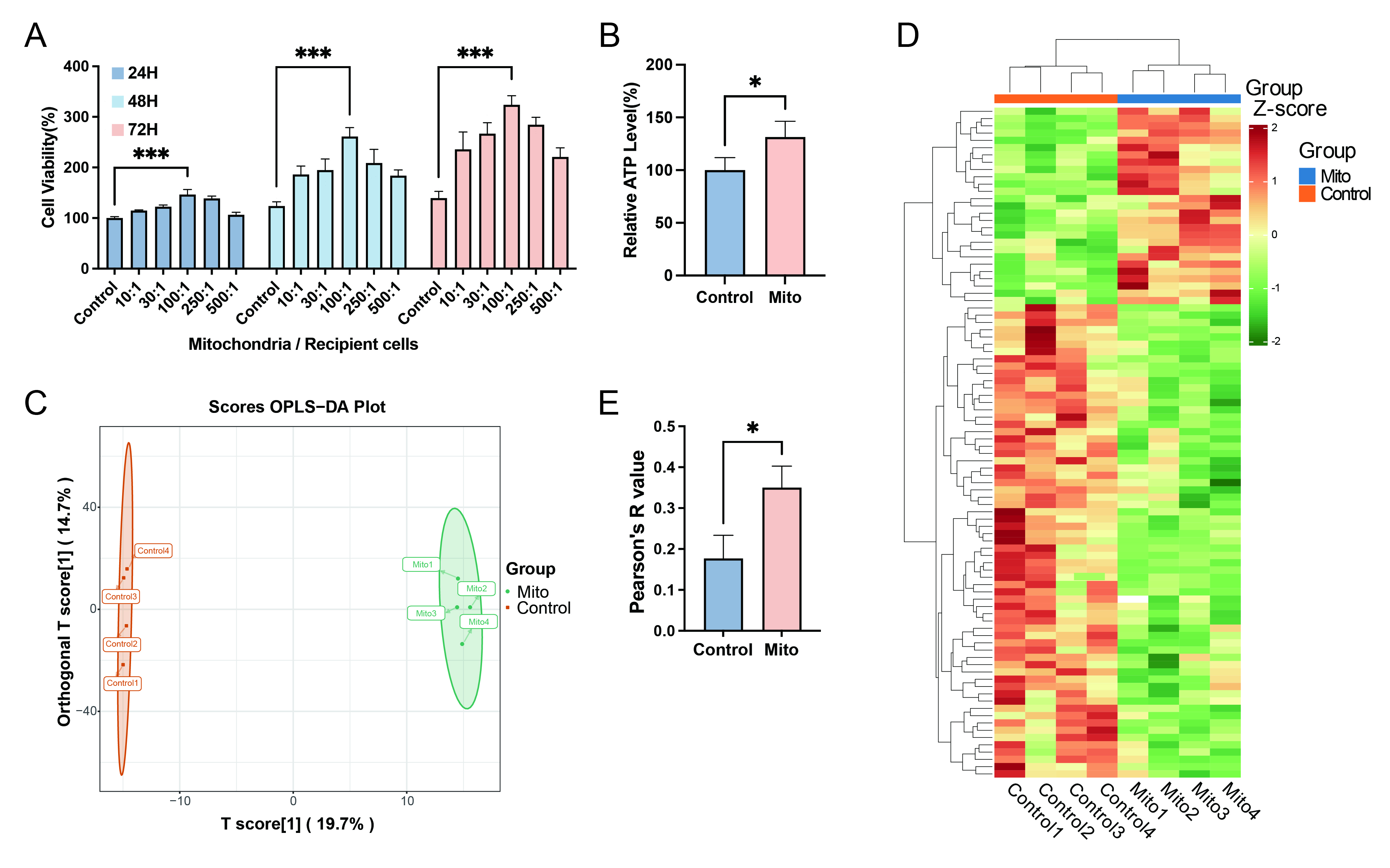


**Figure S1.** A) CCK-8 viability assay of ECs treated with MT at varying mitochondrial number to recipient cell number ratios (control; 10:1; 30:1; 100:1; 250:1; 500:1). B) ATP quantification in ECs after MT at optimal 100:1 ratio. C) Principal component analysis (PCA) of untargeted metabolomic profiles showing distinct clustering of MT-treated vs. control ECs. D) Heatmap analysis of significantly altered metabolites (|log₂FC| > 1, FDR < 0.05) following MT treatment. E) Colocalization analysis of control ECs and MT-ECs. Data are mean ± s.d., n ≥ 3. **p* < 0.05, ***p* < 0.01, ****p* < 0.001.


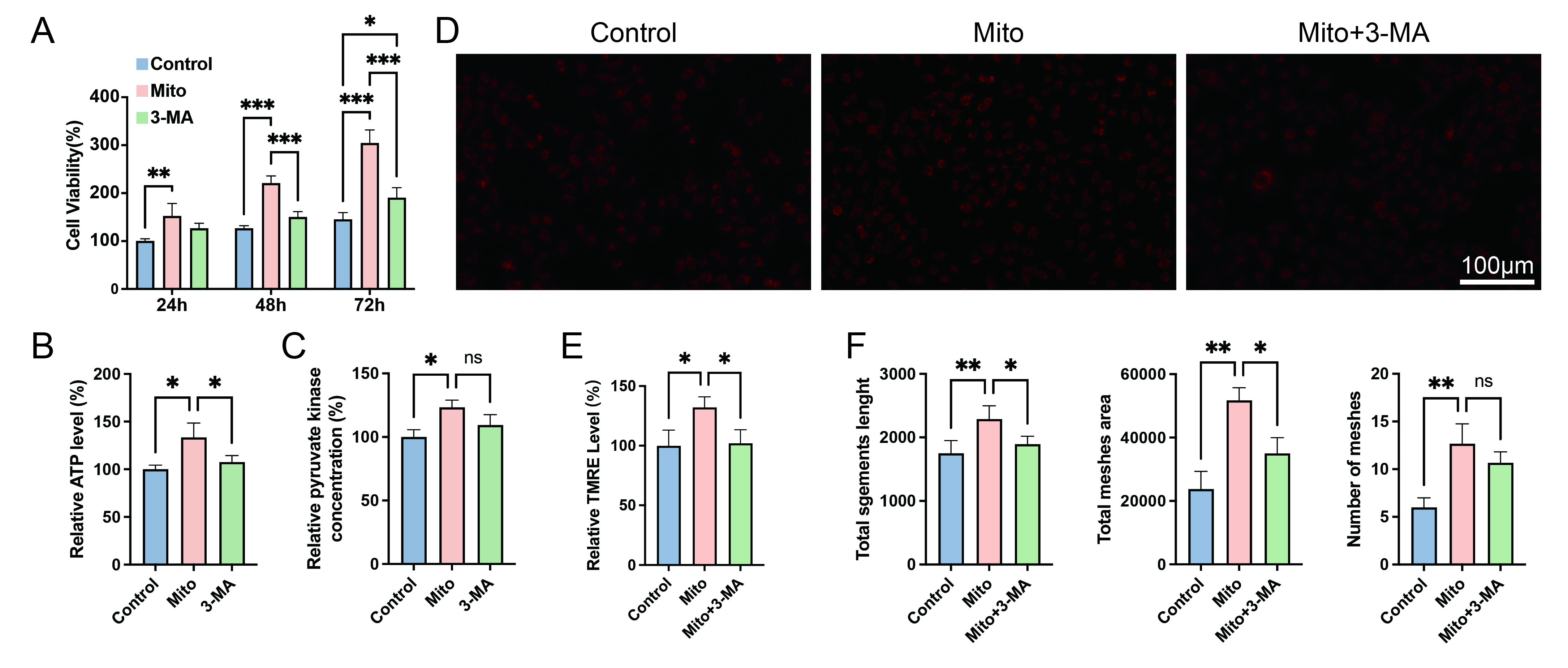


**Figure S2.** A) CCK-8 viability assay of ECs treated with MT or MT+3-MA. B) Quantification of ATP level. C) Quantification of pyruvate kinase concentration. D) Representative TMRE fluorescence images demonstrating mitochondrial membrane potential. Scale bars: 100 µm. E) Quantification of TMRE intensity. F) *In vitro* tube formation assay quantification. Data are mean ± s.d., n ≥ 3. **p* < 0.05, ***p* < 0.01, ****p* < 0.001.


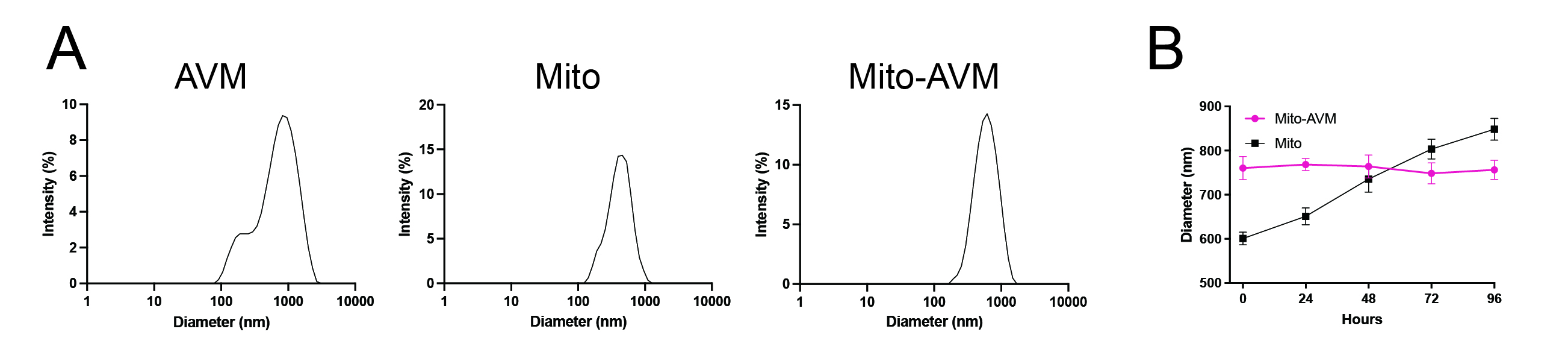


**Figure S3.** A) Hydrodynamic diameter distributions of AVM, Mito, and Mito-AVMs measured by dynamic light scattering. B) Size stability profiles under oxidative stress (100 μM H₂O₂). Data are mean ± s.d., n ≥ 3.


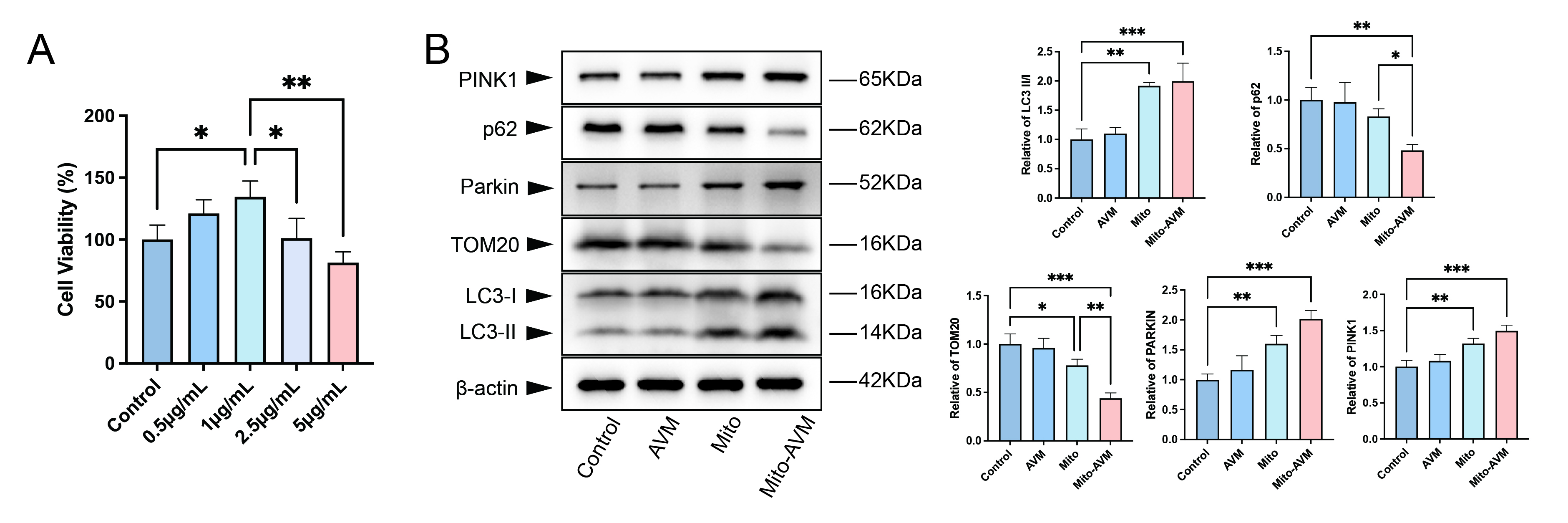


**Figure S4.** A) CCK-8 assay of ECs treated with varying concentrations of AVM for 24 hours. B) Western blot analysis of autophagy markers (LC3-I/II, p62) and mitophagy markers (PINK1, Parkin, TOM20) in diabetic rat wound tissues subjected to four treatments: Control, AVM, Mito, and Mito-AVM. β-Actin served as loading control. Data are mean ± s.d., n ≥ 3. **p* < 0.05, ***p* < 0.01, ****p* < 0.001.


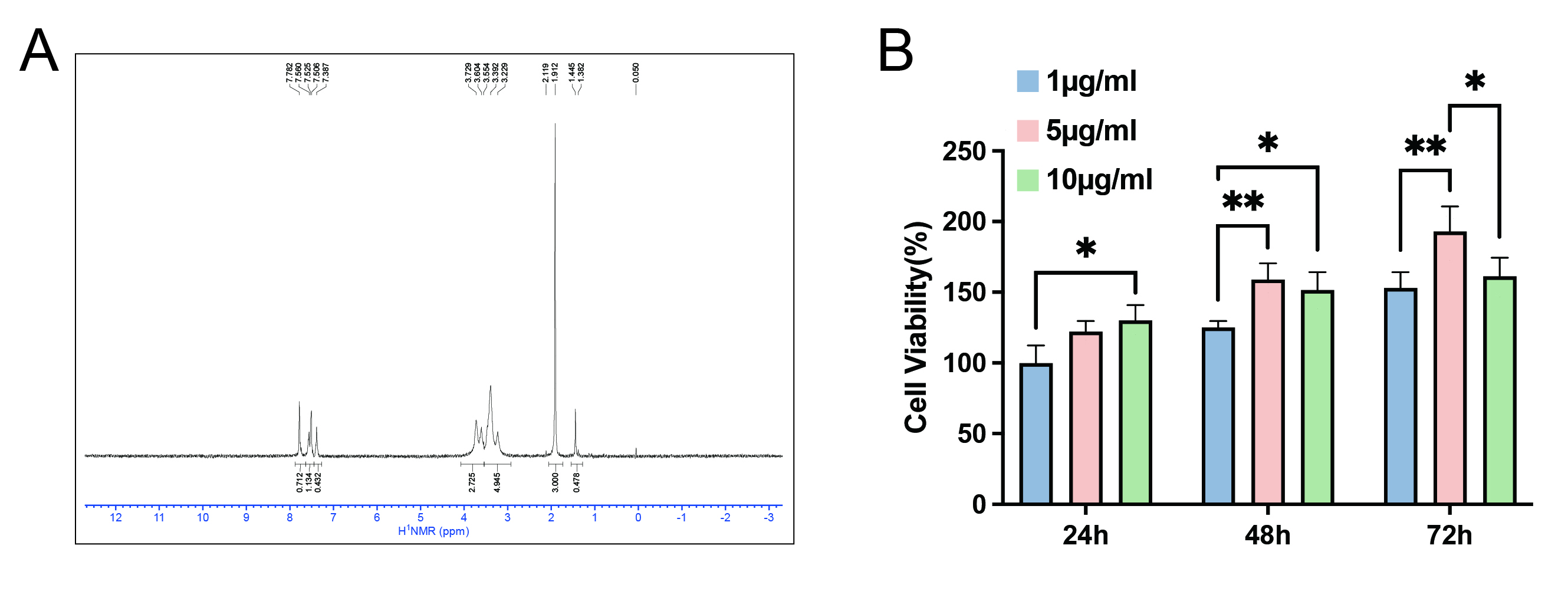


**Figure S5.** A) ¹H-NMR of HA-PBA. B) CCK-8 assay of ECs treated with Mito-AVM@HPP hydrogels at various concentration of Mito-AVM. Data are mean ± s.d., n ≥ 3. **p* < 0.05, ***p* < 0.01, ****p* < 0.001.


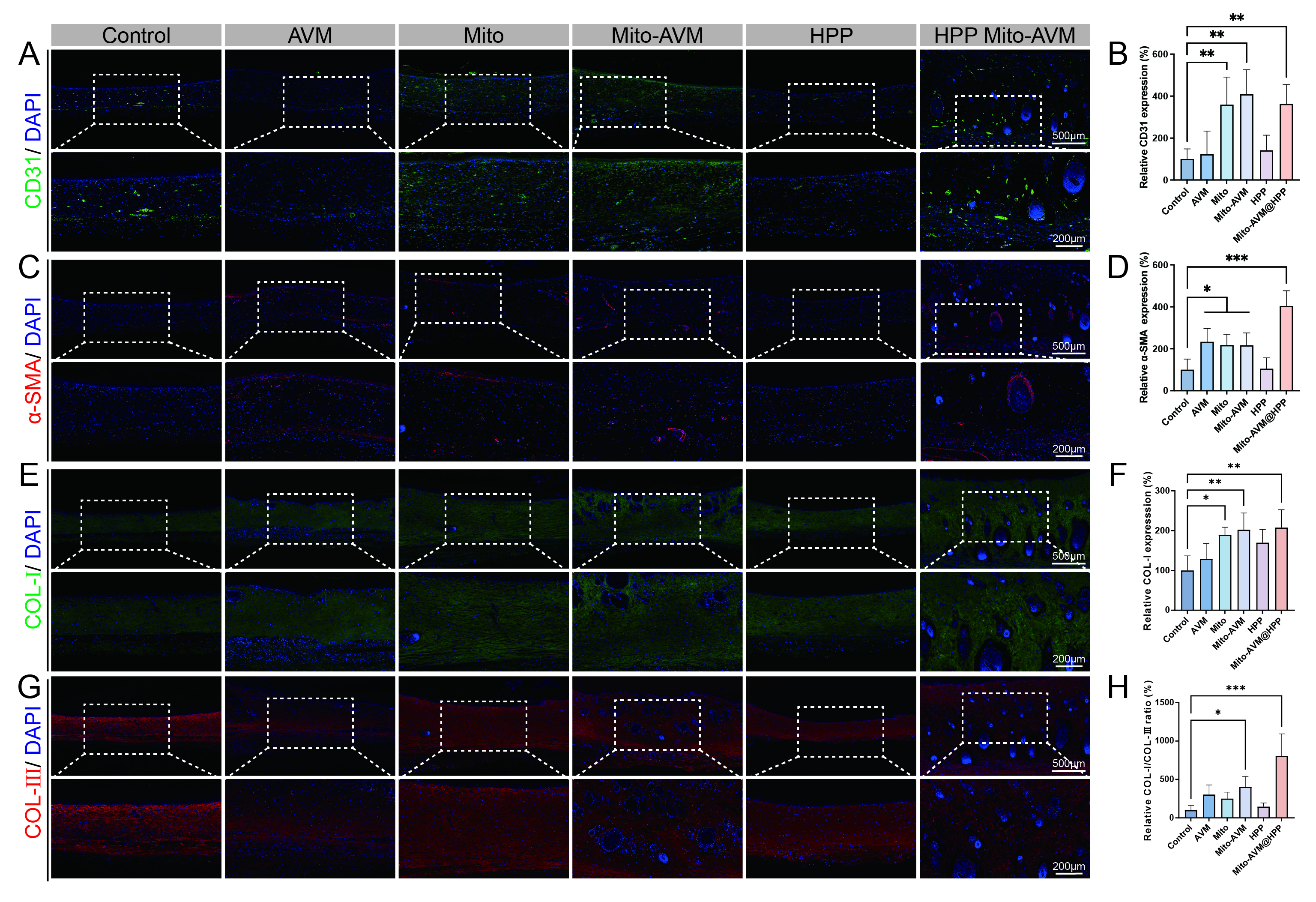


**Figure S6.** Therapeutic Effects on Vascularization and Collagen Deposition in Diabetic Wounds on Day 21. A) Representative immunofluorescence images of CD31. B) Relative CD31 expression. C) Representative images of α-SMA expression. D) Relative α-SMA expression. E) Representative immunofluorescence images of COL-I. F) Relative COL-I expression. G) Representative immunofluorescence images of COL-Ⅲ. (H) Relative COL- I/ COL-Ⅲ expression. Scale bars: 500 µm (main image), 200 µm (magnified view). Data are mean ± s.d., n ≥ 3. **p* < 0.05, ***p* < 0.01, ****p* < 0.001.
