# Supplementary figures and images for: Apoptotic Vesicle Membrane-Mediated Targeted Endothelial Mitochondrial Transplantation-Clearance Therapy for Diabetic Wound Healing
Source: Research (Wash D C). 2026 Jan 16;9:1042. doi: 10.34133/research.1042 (PMC13123279; doi:10.34133/research.1042)

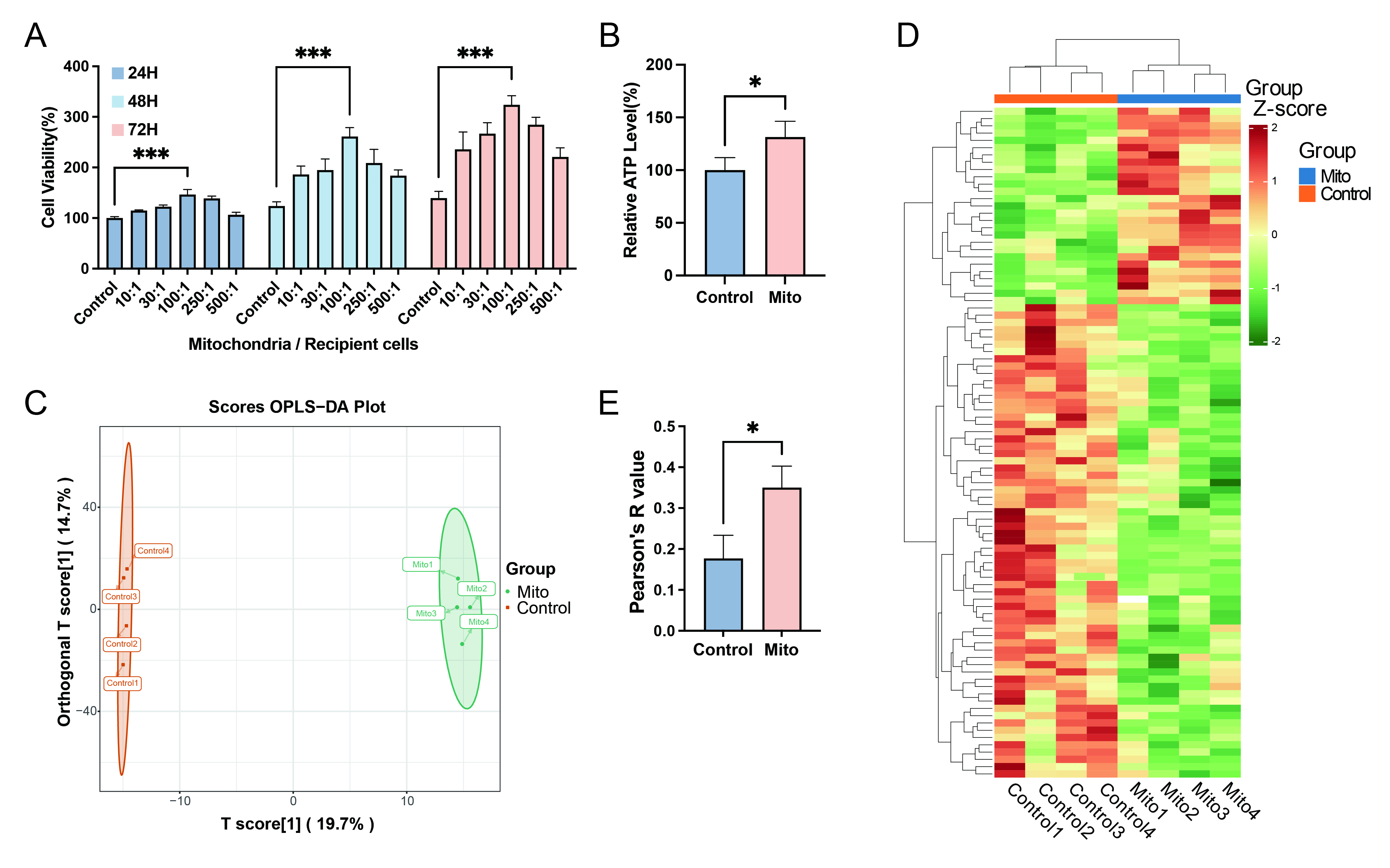

Supplement: Supplementary 1 — Figs. S1 to S6 Movie S1 [file research.1042.f1.zip › SI 1.jpg]

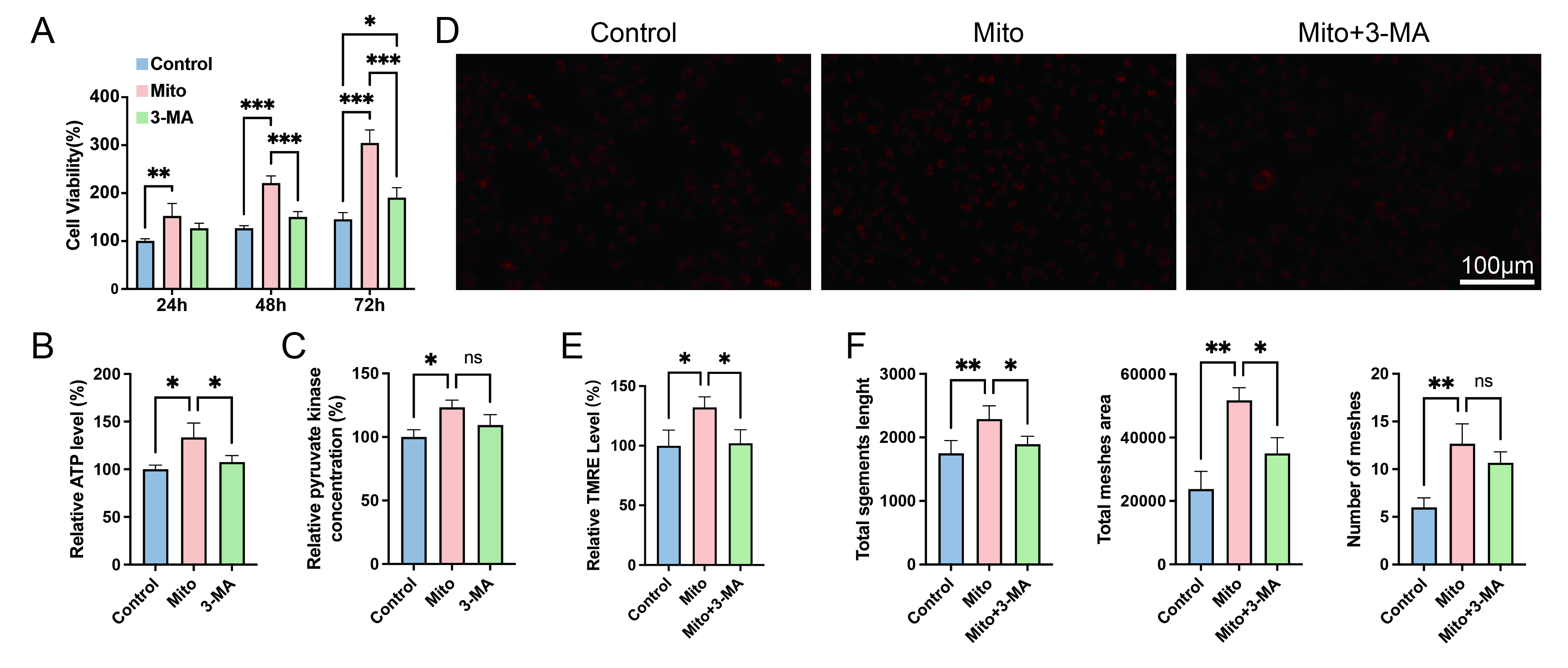

Supplement: Supplementary 1 — Figs. S1 to S6 Movie S1 [file research.1042.f1.zip › SI 2.jpg]

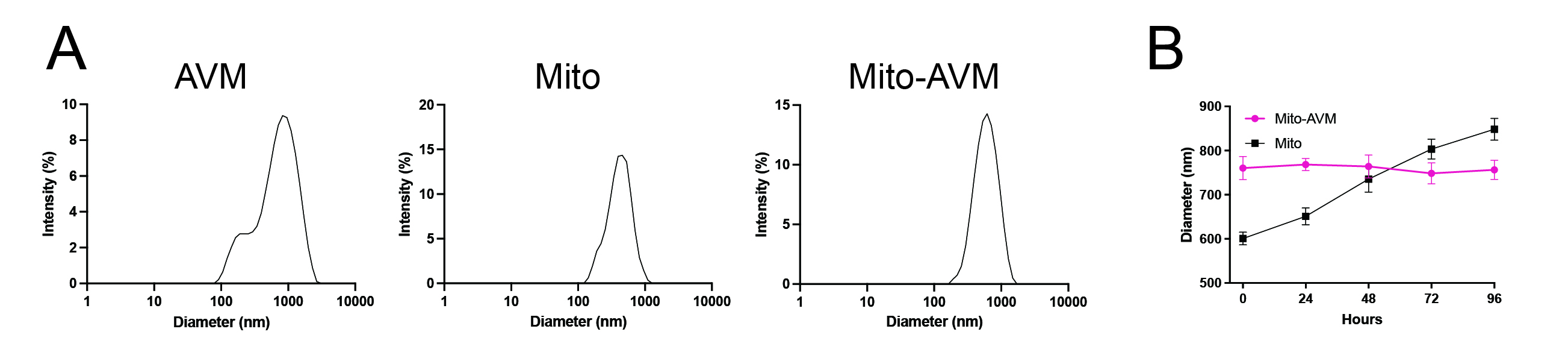

Supplement: Supplementary 1 — Figs. S1 to S6 Movie S1 [file research.1042.f1.zip › SI 3.jpg]

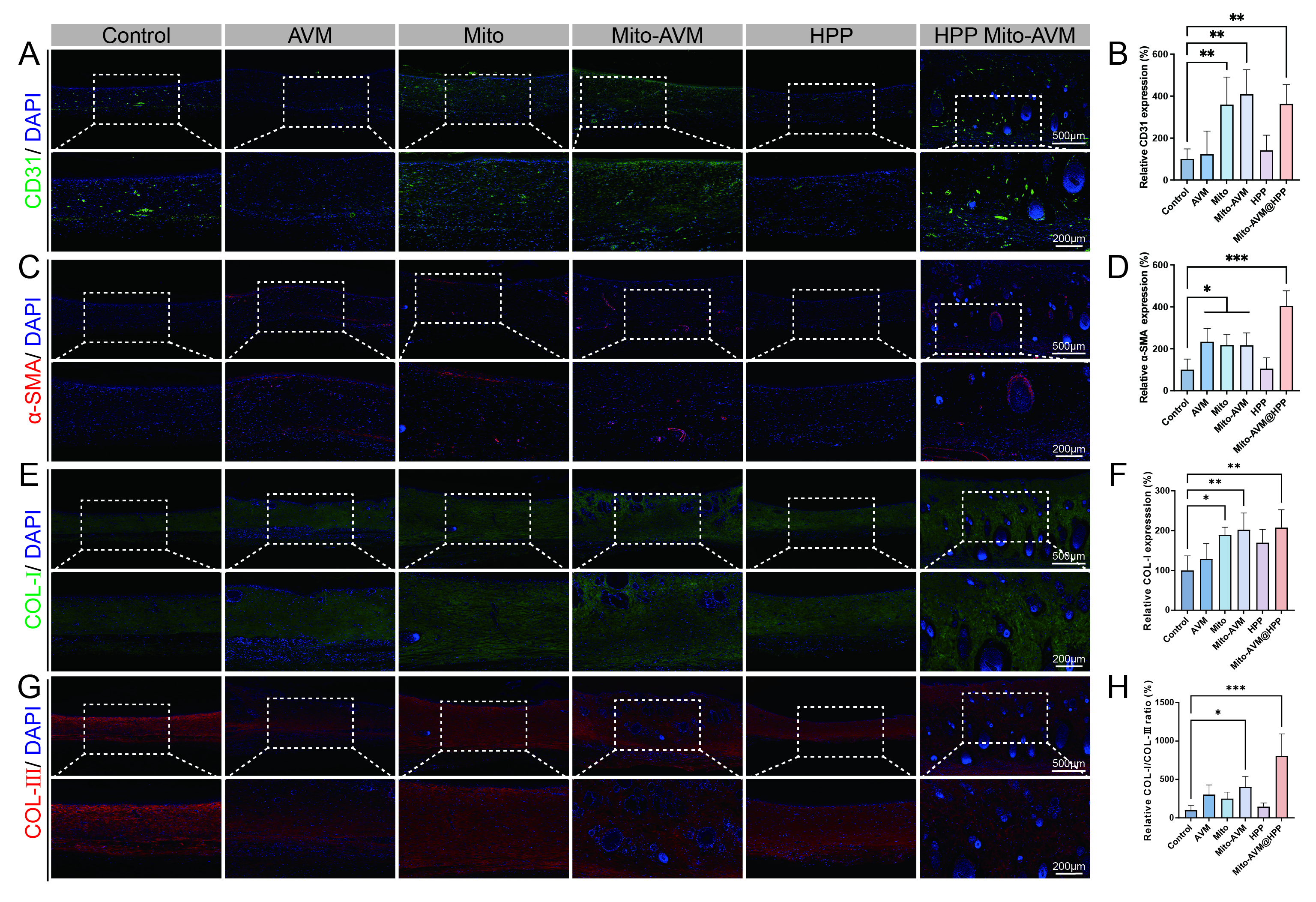

Supplement: Supplementary 1 — Figs. S1 to S6 Movie S1 [file research.1042.f1.zip › SI 6.jpg]

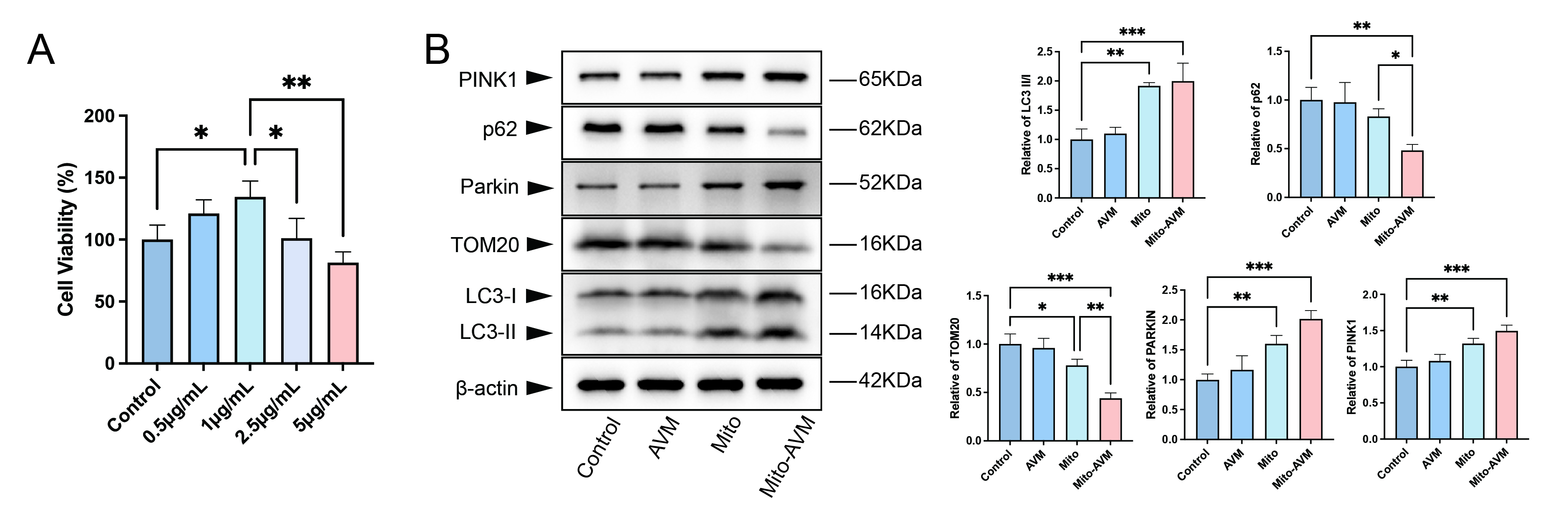

Supplement: Supplementary 1 — Figs. S1 to S6 Movie S1 [file research.1042.f1.zip › SI4.jpg]

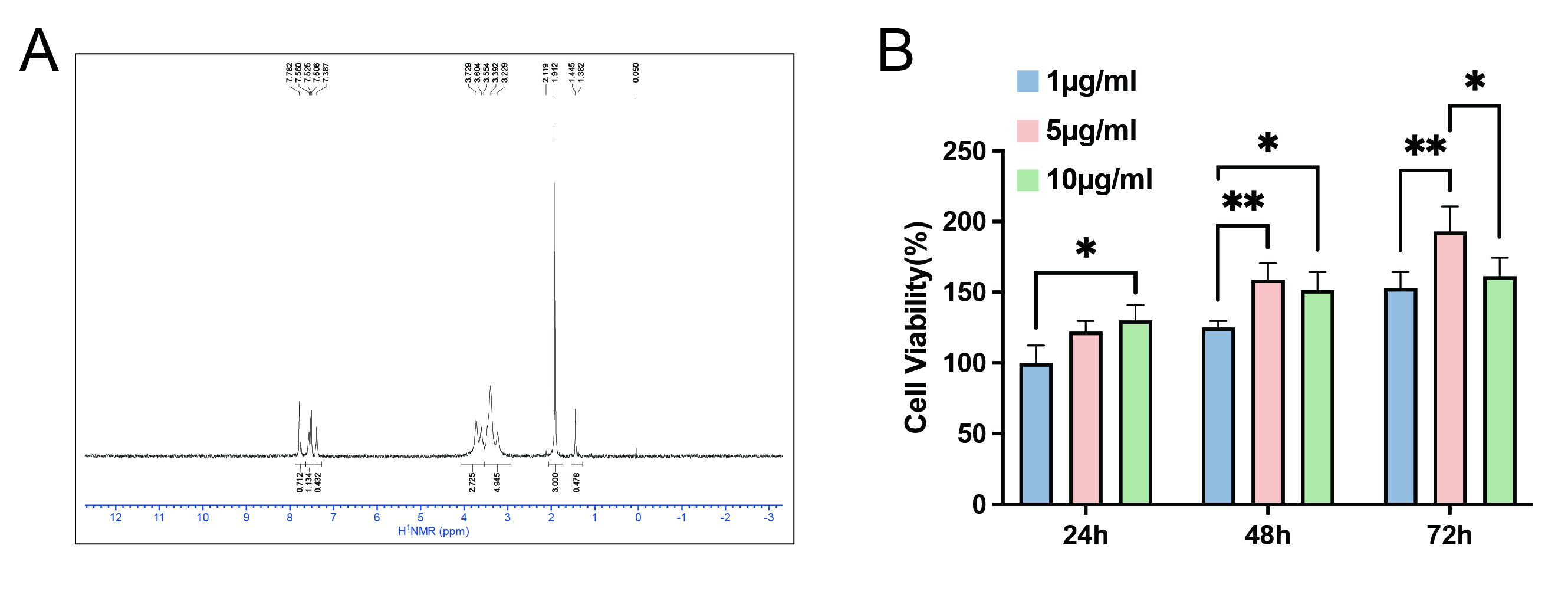

Supplement: Supplementary 1 — Figs. S1 to S6 Movie S1 [file research.1042.f1.zip › SI5.jpg]
